# Selective Recognition of Amino Acids and Peptides by Small Supramolecular Receptors

**DOI:** 10.3390/molecules26010106

**Published:** 2020-12-28

**Authors:** Joana N. Martins, João Carlos Lima, Nuno Basílio

**Affiliations:** LAQV—REQUIMTE, Departamento de Química, Faculdade de Ciências e Tecnologia, Universidade NOVA de Lisboa, 2829-516 Caparica, Portugal; jmn.martins@campus.fct.unl.pt (J.N.M.); lima@fct.unl.pt (J.C.L.)

**Keywords:** amino acids, peptides, host-guest systems, molecular recognition, supramolecular receptors

## Abstract

To this day, the recognition and high affinity binding of biomolecules in water by synthetic receptors remains challenging, while the necessity for systems for their sensing, transport and modulation persists. This problematic is prevalent for the recognition of peptides, which not only have key roles in many biochemical pathways, as well as having pharmacological and biotechnological applications, but also frequently serve as models for the study of proteins. Taking inspiration in nature and on the interactions that occur between several receptors and peptide sequences, many researchers have developed and applied a variety of different synthetic receptors, as is the case of macrocyclic compounds, molecular imprinted polymers, organometallic cages, among others, to bind amino acids, small peptides and proteins. In this critical review, we present and discuss selected examples of synthetic receptors for amino acids and peptides, with a greater focus on supramolecular receptors, which show great promise for the selective recognition of these biomolecules in physiological conditions. We decided to focus preferentially on small synthetic receptors (leaving out of this review high molecular weight polymeric systems) for which more detailed and accurate molecular level information regarding the main structural and thermodynamic features of the receptor biomolecule assemblies is available.

## 1. Introduction

Peptides are fundamental molecules with several biological functions, acting as neurotransmitters, neuromodulators and hormones in numerous biochemical processes, such as quorum sensing, immune response, pain and metabolism, to name a few [1,2]. Given their utmost importance in different biochemical contexts, the discovery of high affinity and selective synthetic receptors for peptide recognition holds a strong potential for enabling new therapeutic agents, such as inhibitors/activity modulators or vehicles for drug delivery, and important molecular components for advanced biological and diagnostic tools [3,4,5,6,7]. Furthermore, the discovery of the fundamental binding interactions controlling the recognition of small peptide sequences is expected to pave the way towards the rational design of synthetic receptors for surface protein recognition. [3,6,8,9,10]. 

However, selective and high-affinity receptors for peptides are recognized to be very difficult to design and synthesize in a rational, bottom-up manner. Being biological molecules, these targets present a difficulty just by the fact that their recognition should be made in water, imposing practical difficulties related to the solubility of synthetic organic receptors, which may require further functionalization to improve their solubility in aqueous solution. Moreover, the solvation of these biomolecules by water and the need to remove the solvent molecules and destabilize the solvation sphere also imposes an energetic penalty that must be overcome upon binding. Directional and strong interactions based on hydrogen bonding, which are frequently and successfully employed in organic solvents, are completely or partially neutralized in aqueous solutions. Therefore, it is necessary to explore other mechanisms, such as the hydrophobic effect and multiple interactions acting cooperatively to achieve the high affinity required to bind the peptide targets at relevant μM concentration or below [11]. In addition, the flexible, rather ill-defined structure of peptide sequences also increases the difficulties associated with the rational design of small receptors [6,12,13,14]. While the challenges associated to the factors enumerated above may be common to other biological targets, peptide recognition, in its completeness, requires receptors that are sequence-selective, discriminating not only the type of amino acids residues composing the peptide but also the order by which the they are arranged. 

Although general methods for high affinity peptide recognition by synthetic receptors are still missing, several research groups have reported encouraging results that show that it is possible to bind specific peptide sequences with simple receptors, such as macrocyclic cavitands, molecular clips, and tweezers or self-assembled coordination cages. Other strategies based on templating methods, such as molecular imprinted polymers and micelles, also showed very promising results. In this review we will cover selected examples of cavitand-based receptors that have been shown to recognize amino acids and peptides in aqueous solution and use these examples to emphasize the noncovalent interactions and geometric factors that control binding stability and selectivity of the different complexes. For this reason, we focus on small molecule receptors for which this type of information can be extrapolated in a more straightforward way and potentially used for the rational optimization of current receptors and design of new ones. Then we will also provide some selected examples of biological and technological applications based on the binding properties of the receptors discussed here to illustrate the potential of these molecules and approaches. 

## 2. The Biological Targets: Amino Acids

Amino acids are the basic building blocks of small peptides and proteins. Even though they are important targets by themselves, the development of supramolecular receptors that can bind specific amino acids with high affinity and selectivity constitutes a first step towards the development of multivalent, sequence specific receptors for peptides. However, the selective binding of free amino acids already presents a difficult task since some side chains of amino acids are very similar to each other. In this section we have divided the 20 common amino acids into four categories: basic, aromatic, acid, and neutral non-aromatic amino acids and discussed their properties. In the next section, their recognition by selected supramolecular receptors will be further discussed. 

### 2.1. Basic Amino Acids 

l-Lysine (Lys) and l-Arginine (Arg) are amino acids that play very important roles in biological systems—these are sites of recognition by the enzyme trypsin [15] and also for methylation in several proteins, as is the case of histones, the proteins involved in the structure of chromatin in the nucleus [16]; both take part in protein–protein binding [17], among other functions [18,19]. Arginine has a very important role in the membrane penetrating properties of antimicrobial peptides, as well [20].

The positive charges on these two amino acids make them easier targets for the development of receptors in water, many of them relying on the formation of several ionic interactions to obtain the receptor-analyte complexes. This is the case of *p*-sulfonatocalix[n]arene [21,22,23], some examples of molecular tweezers [24,25], carboxylatopillar [5]arene [26], all of which have negative charges, to form ionic bonds with the charged amines of Lys and Arg.

*l*-Histidine (His), despite the presence of two nitrogen atoms, is near equilibrium between its deprotonated and protonated form, at physiological pH, due to its p*K*_a_ of 6.5 [27]. Due to this, it can act as a proton donor or acceptor, depending on the environment near the residue [27]. Despite this property, histidine is usually paired with the other two basic amino acids. Regardless, it presents a lower charge density than the other two and thus His is difficult to target selectively by ionic interactions, in favor of Lys and Arg [26,28].

### 2.2. Aromatic Amino Acids

Aromatic amino acids—l-tryptophan (Trp), l-tyrosine (Tyr) and l-phenylalanine (Phe)—are essential for protein function having important roles in protein–protein interaction [29] and electron transport in peptides [30] and proteins [31]. Moreover, these amino acids are precursors of several neurotransmitters—dopamine, serotonin, epinephrine, etc. [32]. Besides these functions, tryptophan also has a role in the ability of antimicrobial peptides to form pores on membranes, through the formation of cation–π bonds with arginine [33].

In terms of protein detection and analytical studies, aromatic amino acids are important because they give proteins the characteristic 280 nm absorption band and are the only amino acids that present fluorescence, specially tryptophan, with a higher quantum yield than the other aromatic amino acids [34]. Many strategies for the recognition of these amino acids are based on hydrophobic, π–π and ion dipole interactions, making use not only of the aromatic group’s characteristic properties but also of the bigger size of these amino acids’ side chain [35,36,37,38,39,40,41,42,43,44,45].

### 2.3. Acidic Amino Acids

The two anionic amino acids l-aspartate (Asp) and l-glutamate (Glu) exert several roles beyond their function as protein monomers. Glutamate is well known for its role as excitatory neurotransmitter in vertebrates, as well as precursor for gamma-Aminobutyric acid, GABA, an inhibitory neurotransmitter [46]. This amino acid is also the precursor to several other biomolecules and takes part in the process of elimination of ammonium, in the urea cycle [47]. Like glutamate, aspartate is also an important precursor in several biosynthetic pathways and takes part in the urea cycle [48]. However, this anionic amino acid is also essential in energetical processes in eukaryotic cells, being part of the malate-aspartate shuttle [49] and also having a role in the gluconeogenesis pathway [50].

Unlike cationic and aromatic amino acids, none of the most used macrocycles or supramolecular systems show particular affinity towards these two amino acids, without the need for further functionalization. Nevertheless, the functionalization of macrocyclic compounds with cationic groups has been demonstrated to yield potential receptors for these amino acids [51,52,53].

### 2.4. Neutral Non-Aromatic Amino Acids

Neutral non-aromatic amino acids are difficult to target selectively in aqueous solution due to their high solvation, similar structures, reduced hydrophobic surface and the lack overall positive or negative charge [3,54]. However, these amino acids still have key roles in proteins and peptides, providing sites for glycosylation [55,56] and other post translational modifications [7]. They are also fundamental in ammonia regulation [57], anabolic and catabolic processes in eukaryotic cells (acting as precursors to other macronutrients or sources of energy) [50] and redox homeostasis [58].

Despite the difficulty of targeting these molecules, some receptors were developed for several of these amino acids. Many of these, however, only reach mM affinities and many were not tested in physiological conditions due to low solubilities in water [54]. Furthermore, many receptors that bind to these biomolecules with higher affinity present very low selectivity and are frequently able to bind to other amino acids with similar or even higher affinities [35,41,43,59].

Thiols are particularly reactive and so, the recognition and detection of l-Cysteine is more commonly based on the creation of sensors that react with this redox biomolecule by nucleophilic substitution [60]. In terms of supramolecular recognition most of the existing systems are based on metal complexes [3].

## 3. Receptors for Amino Acids

Supramolecular chemistry has had a fruitful contribution to the development of new, multifunctional organic molecules. In terms of molecular recognition, a variety of different types of receptors have been developed, from polymeric complex structures to both acyclic and cyclic small molecules [61]. Macrocyclic and semi-rigid receptors have been gaining importance due to their simplicity and, at the same time, high affinity and selectivity towards a myriad of guests, given not only by the complementary noncovalent interactions that can be formed, but also by the size complementarity between host and guest [61,62].

### 3.1. Macrocycles

Macrocyclic receptors are a class of molecules that have a well-defined structure for the binding of a target guest. The cavity in these is often more hydrophobic, while the portals usually present relatively polar or charged moieties [62]. In this section, a select group of examples of macrocyclic receptors, which have shown to bind to amino acids, will be presented.

#### 3.1.1. *p*-Sulfonatocalix[n]arenes

**Figure 1:**
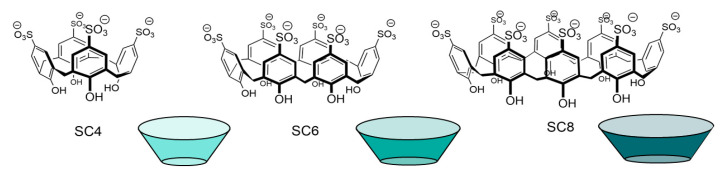
Structures of the *p*-sulfonatocalix[n]arene receptors, SCn, and corresponding schematizations of the macrocycles.

*p*-Sulfonatocalix[n]arene (SCn) macrocycles (Figure 1) were initially synthesized by Shinkai and co-workers to improve the solubility of calixarenes in water and allow for the binding and detection of not only organic compounds, but also inorganic ions in aqueous conditions [21,63,64,65,66,67]. Owing to their high water solubility (>0.1 M) and low toxicity, SCn macrocycles have been widely investigated for their pharmaceutical and biological applications [21,68,69,70,71,72,73,74].

In early studies by Douteau-Guével et al. and Selkti et al. the complexation of *p*-sulfonatocalix[n]arene (n = 4, 6 and 8) with Lys and Arg was explored by NMR spectroscopy and microcalorimetry in solution and by X-ray diffraction in the solid state [21,75,76]. The ^1^H NMR studies carried out at different pH values showed that the calixarenes do not interact with these amino acids at pH 13 due to the deprotonation of the amino and guanidino groups, this last one being only partially deprotonated under these conditions (p*K*_a_ = 13.2) [75]. At more acidic pH conditions (pH 5 and pH 1), the authors also pointed out the weaker interactions between these amino acids and the larger SC6 and SC8 hosts, attributing the low affinity to the more flexible structure of these hosts. It must be noted, however, that the larger SC8 can form 1:1 and 1:2 host:guest complexes with Arg and Lys. SC4 was investigated in more detail at pH 1 and 5, showing that the receptor displays higher affinity for both amino acids at pH 5 and that, independently of the pH, Arg (*K* = 1.7 × 10^3^ M^−1^ at pH 5, *K* = 2.0 × 10^2^ M^−1^ at pH 1) binds more strongly than Lys (*K* = 6.0 × 10^2^ M^−1^ at pH 5, *K* = 1.0 × 10^2^ M^−1^ at pH 1). It was also noted that the presence of high concentrations of metal cations inhibits the formation of the complexes, a phenomenon which was generalized to other guest molecules in later studies and assigned to the competitive binding of the cations (including hydronium) with SCn [77,78,79,80,81,82,83,84,85]. The complexation induced chemical shifts suggests that the terminal amino/guanidino groups in the side chain are included in the cavity of SC4 while the α-amino group remains outside of the cavity, most probably due to repulsion arising from the carboxylate group.

The microcalorimetry studies showed that the binding of both amino acids with SC4 is enthalpy driven (Δ*H*_Lys_ = −14.4 kJ/mol and Δ*H*_Arg_ = −20.3 kJ/mol), with only slight differences in entropy (*T*Δ*S*_Lys_ = 2.0 kJ/mol and *T*Δ*S*_Arg_ = −2.1 kJ/mol), yet with two times higher affinity of the receptor towards Arg than Lys (Table 1), being in line with the previous NMR data [21,75]. This study also confirmed that SC6 and SC8 are in fact weaker binders for these two targets but, contrary to what was reported based on NMR titrations, also suggests that the binding affinity of SC4 increases in more acidic conditions.

**Figure 2:**
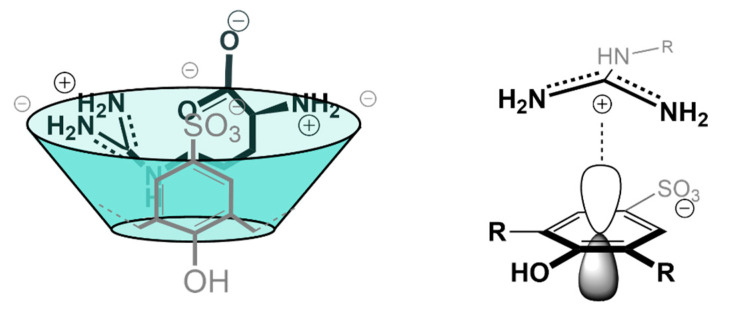
Structure of the complex formed between SC4 and l-Arginine, adapted from [21,71] (left) and a schematization of the cation–π interactions that are established in the former, between the aromatic rings of the receptor and the guanidinium moiety of the analyte.

**Figure 3:**
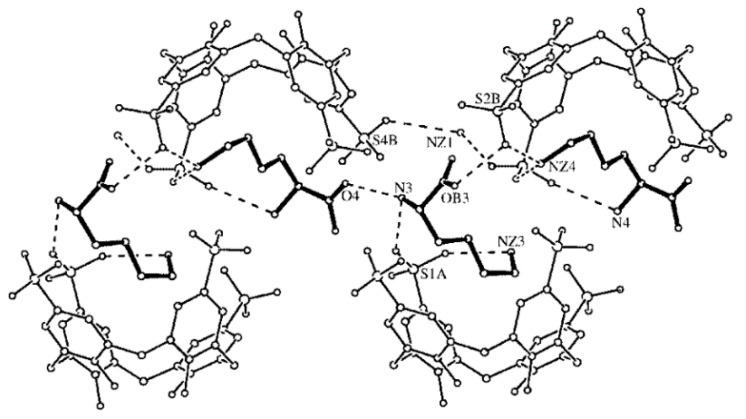
Crystallographic structure of the SC4-l-Lysine: the analyte (in bold) complexes in a parallel position to the calixarenes, as it can be observed by the two top complexes; another possible conformation is perpendicular to the disposition of the calixarenes, interacting not only with the calixarene at the bottom but having its ε-amino group, NZ1, interacting externally with the sulfonate groups of the top layer receptors. Reprinted with permission from [76]. Copyright (2000) The Royal Society of Chemistry.

The thermodynamic information given by the microcalorimetry studies indicates that the more favorable enthalpy of the Arg complex is probably due to the formation of cation–π and π–π interactions between its guanidinium group and the aromatic rings in the cavity of the calixarene (Figure 2). Moreover, this further stabilization can justify the negative value of entropy, seeing as arginine binds deeper in the cavity of the receptor [71] and so, with reduced degrees of freedom [21]. The affinity of this receptor towards l-Histidine (His) was also tested, however, the affinity obtained was two times lower than towards Lysine and near the affinity towards neutral amino acids [28]. In addition to the studies made in solution, the crystallographic structures of the Lys (Figure 3) [76] and Arg [71] complexes further support the recognition of these amino acids through the inclusion of their side chains in the cavity of the SC4 receptor.

Several more recent studies also measured the affinity of these complexes, using indicator displacement assays, in different conditions, which are summarized in Table 1. Many of these yielded higher affinities of the cationic amino acids towards the receptor SC4, due to this smaller macrocycle having a more rigid structure in comparison to SC6 and SC8. These studies also indicate that, near physiological conditions, the binding may vary, considering that the presence of other cationic molecules, such as buffer counterions, can interfere extensively with the binding [21,75,86,87,88].

**Figure 4:**
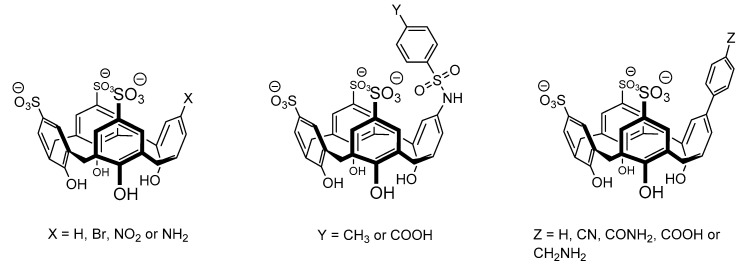
Structures of the upper rim monofunctionalized sulfonatocalix[4]arene receptors synthesized by Hof, where the structure with an added aromatic ring with Z = H, corresponds to SC4-Ar [90].

The higher affinity of SC4 towards methylated lysine is also worth noting, an important post-translational modification. Hof and co-workers observed that the affinity of the 1:1 complexes formed by SC4 with lysine and its methylated derivatives increases with the degree of methylation reaching ca. 70-fold selectivity to trimethyllysine over lysine [89]. Based on NMR titration experiments, the authors observed that while the lysine side chain is accommodated to the SC4 pocket in a “side-on” binding mode, in trimethyllysine the NMe_3_^+^ group is deeply included in the cavity suggesting an optimal charge and shape complementary between this group and the calixarene receptor. In a following example, the same research group explored the monofunctionalization of trisulfonated calix[4]arenes (Figure 4) to tune their affinity and selectivity towards trimethyllysine. Although most of the different receptors showed lower binding affinities, SC4-Ar, the one with the unsubstituted aromatic panel (Z = H) directly connected to the calixarene rim, was found to display the higher binding affinity towards trimethyllysine, almost 2-fold larger than the one previously observed for SC4 and an improved selectivity of 150-fold over unmethylated lysine (Table 1). This is presumably due to its higher hydrophobic character and additional CH-π interactions between the lysine side chain and the extra aromatic wall [90].

#### 3.1.2. Pillar[n]arenes

**Figure 5:**
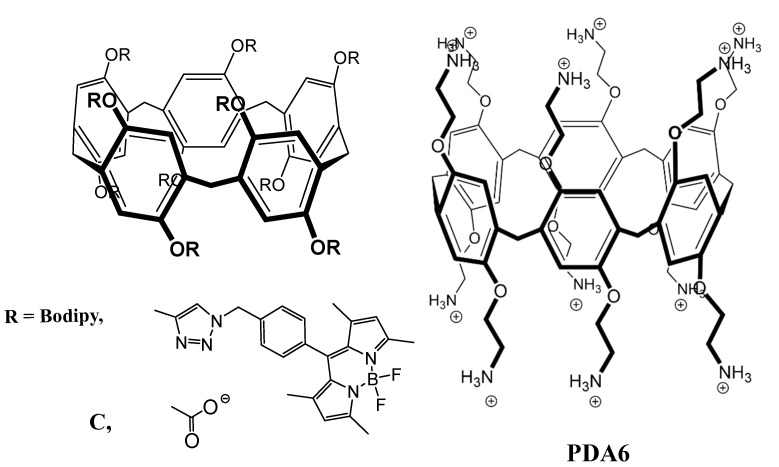
Structure of the Pillar[5]arene receptors, P5-Bodipy [95], carboxylatopillar[5]arene, C-P5 [26], and dodecaamine pillar [6]arene, PDA6 [51].

Pillararenes are a relatively new class of macrocycles, that only recently have begun to be applied for the binding of analytes in water [91,92,93]. Carboxylatopillar [5]arene (CP5) was one of the first water-soluble pillararenes to be synthesized [91] and recently it has been applied as a receptor for cationic organic molecules. Similar to SCn, these molecules present a rigid electron rich cavity lined with anionic groups on both sides of the cavity (Figure 5) [26,94].

In line with its ability to bind positively charged guest molecules, CP5 was demonstrated to selectively bind basic amino acids, i.e., l-lysine, l-arginine and l-histidine, with mM affinity (Table 1). In contrast with SC4, the biomolecules penetrate completely into the cavity, with the amine group of the chiral center being stabilized by ionic interactions at one of the entrances, and the amine in the side group of the amino acid being stabilized by the opposite side. Although these interactions have a bigger role in stabilizing these complexes—as suggested by the selectivity towards cationic amino acids observed in the study—the hydrophobic effect also takes an important role, namely between the CP5 cavity and the aliphatic chains of the amino acids. Furthermore, CP5 has an even higher affinity towards arginine over the other two cationic amino acids, due to the presence of the guanidinium moiety. This group allows for cation–π interactions with the π orbitals of the cavity of the pillararene, as well as the possibility to form several H-bonds with the receptor due to the conjugation between the two nitrogen atoms [26,94].

Another example of a pillararene receptor, is dodecaamine pillar [6]arene, PDA6, which has 6 amine groups at each of its entrances (Figure 5) and has been shown to bind to acidic amino acids [51]. The principles behind the affinity of PDA6 towards these two amino acids is the same as for CP5—the amine groups are positively charged at physiological pH and they can establish ionic interactions with the two carboxylic groups of both Glu and Asp. Although this is the main driving force of the binding (with affinity in the μM range) the complex can be further stabilized by cation–π interactions between the cavity of the pillararene and the amine group of the amino acids. This not only adds attractive interaction between the molecules, but also counterbalance the electrostatic repulsion that could be stablished between the cationic regions of both receptor and analyte [51].

Pillararenes can be applied for the recognition of neutral amino acids as well. This has been explored by Guler and co-workers [95], who developed a pillar[5]arene-based sensor, P5-Bodipy, which presented high selectivity towards l-Asparagine. l-Asn is an analogue of l-Aspartate, with an amide group in its side chain instead of a carboxylate group. Due to its zwitterionic character and neutral side chain, the influence of electrostatic interactions between receptor and analyte on the receptor’s selectivity is negligible, being mostly defined by the hydrogen bonds, hydrophobic and Van der Waals interactions [54,95]. P5-Bodipy is composed by a pillar[5]arene macrocycle, decorated with BODIPY moieties (Figure 5). This receptor detected l-Asn selectively by fluorescence spectroscopy, showing an increase in the Bodipy fluorescence. ^1^H-NMR elucidated the types of interactions present, showing that l-Asn is inserted in the pillararene cavity, being stabilized by several types of electrostatic interactions, but with Van der Waals interactions and hydrogen bonds being the most relevant for the selectivity of the receptor.

#### 3.1.3. Cucurbit[n]urils

**Figure 6:**
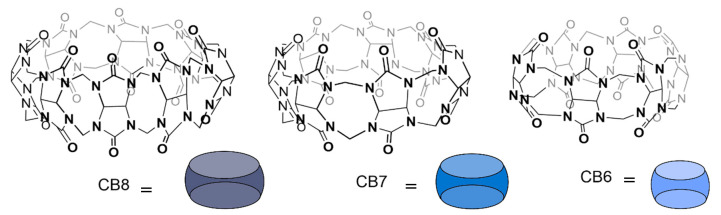
Structure of the Cucurbit[n]uril (CBn) receptors and corresponding schemes that will be used for illustration purposes throughout this review.

Cucurbit[n]urils (CB[n]) are macrocycles composed of n glycoluril units, that, owing to their outstanding binding properties, are largely applied for the recognition of several organic molecules and biological analytes (Figure 6) [44,96,97,98,99,100]. Thanks to their hydrophobic cavity and highly electronegative carbonyl portals, these macrocyclic containers are particularly suitable to bind positively charged and hydrophobic guests. Buschmann et al. [35] performed earlier studies on the complexation of amino acids with CB6 which showed mM affinity towards the Phe, Gly, Ala, and Val, in aqueous formic acid 50%(*v*/*v*). Nau and co-workers reported the complexation of lysine, arginine and histidine by CB7 with all three amino acids showing moderate affinity in the mM range [45]. Later on, Macartney et al. investigated the recognition of lysine, arginine and their methylated derivatives by CB6 and CB7 [101]. Noteworthy, the affinity increases significantly upon lysine methylation, reaching a 3500-fold selectivity for trimethyllysine over lysine as a result of the good complementarity of CB7 and trimethylalkylammonium motifs. On the other hand, at neutral pH, lysine seems to bind only in the CB6 portal, forming an exclusion complex that, nevertheless, is more stable than the one formed with the larger CB7 (1.1 × 10^4^ M^−1^ vs. 5.2 × 10^2^ M^−1^). A subsequent systematic study on the formation of host–guest complexes between CB7 and different amino acids revealed that their stability increases under acidic conditions [102]. The differences in association constants obtained at different pH values are particularly relevant for lysine and arginine which show ca. 3 order of magnitude increases from ≈10^2^ M^−1^ at neutral pH to ≈10^5^ M^−1^ at pH 2. This observation puts in evidence the repulsive interactions established between the carbonyl portals of the receptor and carboxylate group of the amino acid. This destabilizing factor is more relevant in these two cases, most probably due to the fact that their side-chain must penetrate deeply into the CB7 cavity for optimal binding interactions, which are counteracted by the negatively charged carboxylate groups at neutral or slightly acid conditions [102].

**Figure 7:**
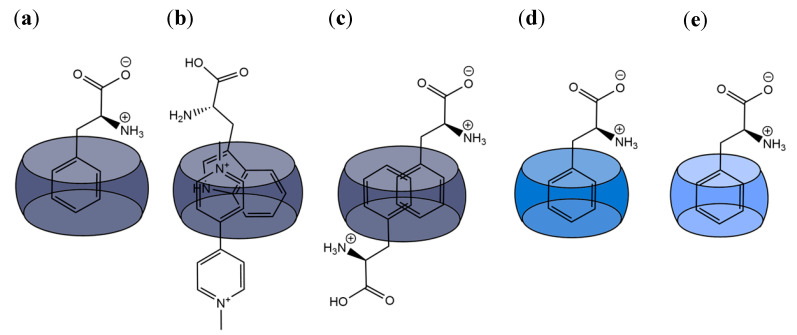
Examples of the CB[n] complexes: (**a**) CB8’s complex with one Phe molecule; (**b**) CB8’s heteroternary complex with Trp and Methyl Viologen; (**c**) CB8’s homoternary complex with two Phe molecules; (**d**) CB7 complex with Phe; (**e**) CB6 complex with Phe.

Despite being investigated for their affinity towards basic amino acids, CB7, CB8 and other CB[n] derivatives were found to be more successful at targeting aromatic amino acids in biologically relevant conditions [44,102]. Among the different CB[n] homologues, CB6 was shown to display weak affinity towards aromatic amino acids probably due to the small size of its cavity (Figure 7e), while the ones with more than 6 glycoluril units have a large enough cavity to accommodate the aromatic side chain in its interior [36,37,38,39,44,45]. This is the case of both CB7 and CB8 that were reported to, respectively, form 1:1 complexes and 1:1/1:2 complexes with aromatic amino acids (Figure 7a,c,d) [36,37,38,39,45].

CB7, for example, was shown to be selective for aromatic amino acids at pH 7 (see Table 1) displaying higher affinity for l-phenylalanine with binding constants in the order of 10^5^ M^−1^–10^6^ M^−1^ depending on the medium conditions, in particular on the presence of salts [102,103,104]. Amongst the different aromatic amino acids, CB7 is selective for Phe by a factor of approximately 10 over Tyr and by a factor of 100 over Trp [102]. Most thermodynamic studies show that the complexation process is enthalpy driven in great part due to the displacement of high energy water molecules from the cavity of the host [102,105,106,107,108]. This hydrophobic effect can be complemented by attractive ion-dipole interactions between the protonated amino group of the biomolecules and the carbonyl group of the cucurbituril (Figure 7) [35,44,109]. In the case of the binding of aromatic amino acids by CB7, the process is also enthalpy driven with unfavorable negative entropy contributions. The enthalpic gain is only slightly higher for phenylalanine with respect to tyrosine and tryptophan, but the entropic loss is very low in first case (*T*Δ*S* = −0.6 kJ.mol^−1^) becoming more important for tyrosine (*T*Δ*S* = −3.7 kJ.mol^−1^) and tryptophan (*T*Δ*S* = −11.3 kJ.mol^−1^) [102]. On the basis of these thermodynamic parameters, the observed selectivity trend for recognition of aromatic amino acids by CB7 was explained by taking into account the higher hydrophobic character of phenylalanine comparatively with tyrosine and the restricted motion of the indole side chain of tryptophan inside the CB7 cavity [102].

CB8, on the other hand, binds aromatic amino acids with 1:1 (Figure 7a) and 1:2 (Figure 7c) stoichiometry, as mentioned above [110]. The ITC binding studies performed on these systems did not allow the determination of the stepwise binding constants and thermodynamic parameters for the 1:1 and 1:2 complexes in separate which precludes a more in depth thermodynamic analysis. Nevertheless, based on the obtained overall binding constants (i.e., *K*_1:1_*K*_1:2_) one can conclude that CB8 is also more selective for phenylalanine, but in contrast with CB7, tryptophan is complexed with only slight lower affinity than phenylalanine while the affinity of tyrosine seems to be too low to be measured [110].

The larger cavity of CB8 allows the binding of amino acids in the presence of auxiliary guests based on electron deficient organic molecules, such as methyl viologen (MV) [37], 2,7-dimethyl- diazaphenanthrenium (DPT) [38] and tetramethyl benzobis(imidazolium) (MBBI) [39]. The preformed 1:1 CB8:auxiliary guest complex can be viewed as a new receptor capable of forming 1:1:1 heteroternary complexes with electron rich molecules that, in some cases, does not bind significantly to CB8 alone (Figure 7b) [37,38,39]. Interestingly, the CB8:MV complex was shown to form 1:1:1 complexes only with the aromatic amino acids (except histidine). In the 1:2 complex, phenylalanine establishes strong contacts with the second Phe residue inside the cavity, as well as with CB8 itself [110]. Differently from what is observed for CB8 in the absence of auxiliary guest, the CB8:MV supramolecular receptor shows higher affinity for tryptophan (4.3 × 10^4^ M^−1^) than for phenylalanine and tyrosine [110]. This selectivity was attributed to the charge transfer interactions between MV and the indole side chain of this amino acid inside the CB8 cavity. The nature of the auxiliary guest may also influence the recognition of the second guest. CB8:MBBI displays binding affinities for tryptophan (3.4 × 10^4^ M^−1^) [39] similar to the one observed for CB8:MV, while CB8:DPT forms 1:1:1 complexes with this amino acid that are one order of magnitude more stable (4.2 × 10^5^ M^−1^) [38].

**Figure 8:**
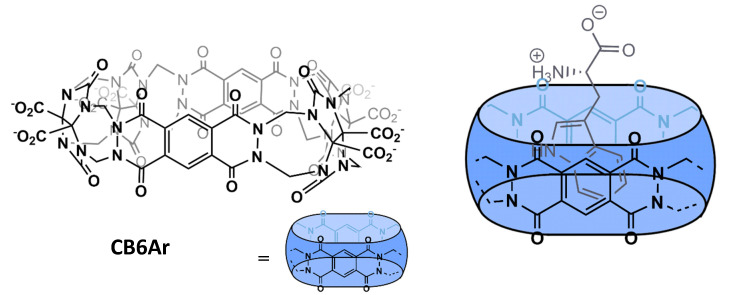
Structure of the modified CB6 receptor, CB6Ar, and a schematization of the complex formed between the receptor and l-Trp, with the establishment of two π–π interactions [40].

Cucurbiturils can also be modified covalently to enhance their affinity towards amino acids. One example of this is the work done by Isaacs and co-workers, which incorporate two aromatic rings within the CB6 macrocycle. This study yielded a water-soluble receptor with an elongated cavity, CB6Ar (Figure 8), which is able to form π–π interactions with included analytes, while maintaining CB[n]’s capacity to form ion-dipole interactions at the macrocycle entrances. The addition of the π–π interaction renders a receptor with micromolar affinity towards tryptophan (*K*_a_ = 3.2 × 10^6^ M^−1^) and ca. 100-fold selectivity for this amino acid over phenylalanine and tyrosine [40].

#### 3.1.4. Cyclodextrins

**Figure 9:**
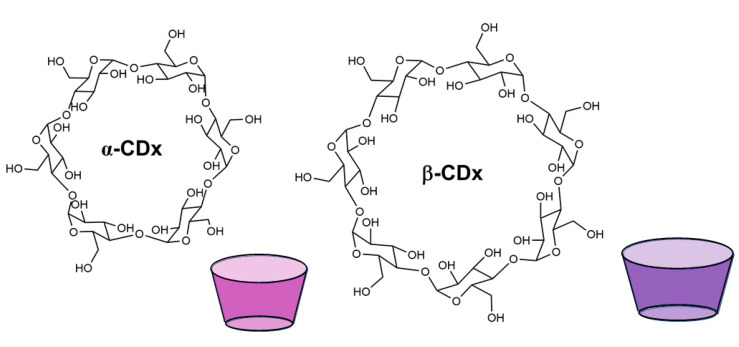
Structure of the receptors α-Cyclodextrin (α-CD) and β-Cyclodextrin (β-CD) and correscheme 41. recently synthesized a single substituted analogue of this macrocycle, β-CDU, with high affinity towards l-tryptophan (*K* = 5.2 × 10^4^ M^−1^), thanks to the added “arm” with hydrogen bonding groups as well as a hydrophobic region (Figure 10). Despite the larger cavity of β-cyclodextrin, structural studies showed that there was no inclusion complex, contrary to what is usual for these systems, but instead the complex was formed at the entrance of the receptor. This binding was shown to happen in 2:1 host:guest stoichiometry with the receptor forming a dimer, with the analyte in the created pocket, being stabilized by hydrophobic interactions and hydrogen bonds [41].

Cyclodextrins (CD) are macrocycles formed by glucose units that can bind a variety of different small molecules. The most common ones are those composed of 6, 7, and 8 glucose units, corresponding respectively to α-CD, β- CD, and γ-CD (Figure 9) [111]. Their host–guest complexes with biomolecules are usually low in specificity, but are slightly more stable with aromatic or small, less polar molecules

α-cyclodextrin was shown to bind l-Phe in a 2003 study by Buschmann et al., but the binding is rather unspecific, only driven by hydrophobic interactions, yielding the same affinities for this amino acid and for a series of other less polar amino acids, as well as other non-polar analytes tested [35].

#### 3.1.5. Cavitands

**Figure 11:**
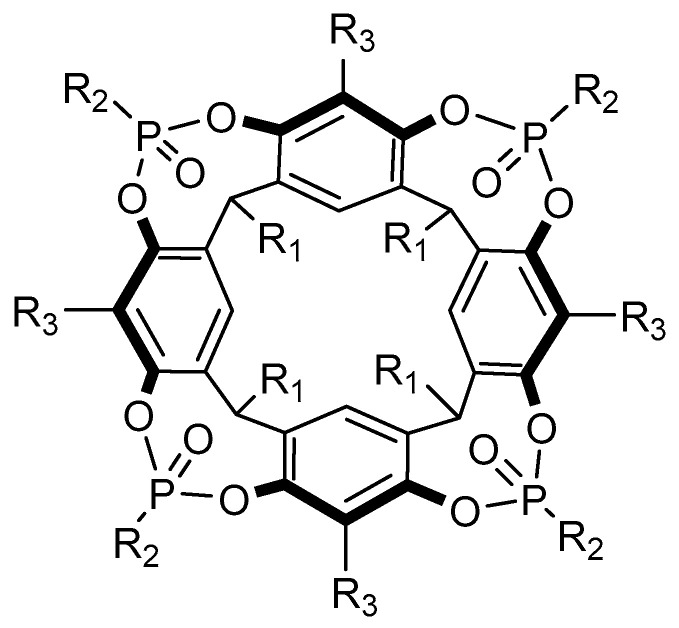
General structure of the Dalcanale cavitand receptors, Cav[R_1_,R_2_,R_3_] [113,114,115].

Cavitands is a term that encompasses several types of rigid macrocycles and it was first coined by Cram and co-workers in 1982 [112]. These constitute a class of receptors that present a “enforced cavity” where a small guest can bind, being the most widely studied ones those based on resorcinarenes macrocycles [62,112]. The Dalcanale group has reported several cavitand derivatives (Figure 11) that were found to recognize amino acids and amino acid metabolites [113,114], and were able to obtain the crystallographic structure of the complexes between some of these cavitand receptors and all basic amino acids, Arg, Lys, and His. The 1:2 complexes were shown to be mainly stabilized by direct and water-mediated hydrogen bonds between a guest molecule and two host molecules [114]. These receptors were also shown to be particularly adequate to selectively recognize sarcosine, a potential prostate cancer biomarker, over glycine in water and in biological fluids [113]. The crystal structures of the complexes showed that while glycine methyl ether is localized at the receptor’s portal, sarcosine forms an inclusion complex with the *N*-methyl group deeply included in the cavity. This structural arrangement allows the stabilization of the complex by a combination of non-covalent interactions that include CH-π contacts between the *N*-methyl residue and the aromatic pocket, along with cation-dipole and hydrogen bonding between the ammonium and the phosphonate groups. The selective complexation of sarcosine in solution was confirmed by ITC experiments in methanol, by water:chloroform extraction and solubilization assays. The recognition of sarcosine in water and in urine was achieved by grafting the cavitands to a silicon surface. The authors showed that the receptor held its recognition properties at these interfaces and devised a sensor based on a luminescence indicator displacement assay using (9-anthrylmethyl) methyl ammonium chloride as probe.

More recently, a comprehensive study on recognition of amino acids by tetraphosphonate cavitands was performed to dissect the thermodynamic factors responsible for the observed selectivity of these receptors towards *N*-methyl amino acids [115]. The complexation was investigated both in methanol and in aqueous solutions, showing that the stability of the resulting complexes significantly decreases (almost 2 order of magnitude) in water. The ITC results demonstrated that while the association process is both enthalpy and entropy-driven in methanol, it becomes entropically unfavorable in water. The authors attributed this observation to the non-classical enthalpy driven hydrophobic effect and to the enhanced solvation of the complex in aqueous solution. Despite of the decreased affinity, the selectivity of these receptors toward *N*-methylated amino acids improves in aqueous solution resulting in their exclusive complexation.

#### 3.1.6. Other Macrocyclic Receptors

A variety of different macrocycles have been used for the binding of a myriad of molecules. Some less known examples that are only now being applied in biomolecule recognition or that present more complex molecular structures are described below, showcasing the versatility and diverse design that macrocycles can present.

**Figure 12:**
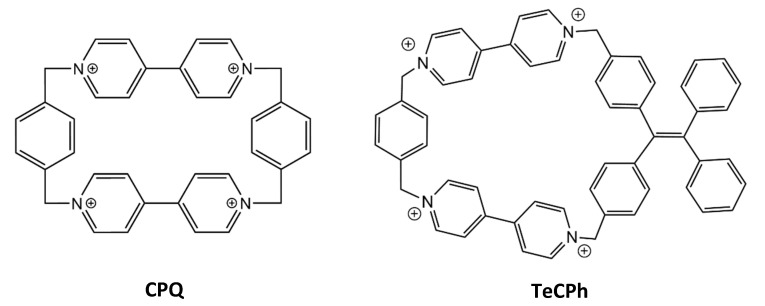
Structure of the cyclophane receptors CPQ [116] and TeCPh [42].

Early work by Kaifer et al. showed that the tetracationic cyclophane cyclobis(paraquat-p-phenylene), CPQ, commonly known as the blue-box, forms charge transfer complexes with aromatic amino acids in aqueous solution (Figure 12). This receptor is selective for tryptophan (*K* = 1.0 × 10^3^ M^−1^) showing ca. 2 and >10 fold lower affinity for tyrosine and phenylalanine, respectively [116]. Cao and co-workers more recently explored the affinity of two distinct tetracationic cyclophane derivatives towards anionic and aromatic biomolecules. The TeCPh, the cyclophane analogue with a narrower electron-deficient cavity described in this study (Figure 12), showed a high specificity towards tryptophan (*K* = 1.2 × 10^3^ M^−1^) in relation to the other amino acids, forming π–π bonds with the electron-rich aromatic side chain of the analytes, and possibly being further stabilized by ionic interactions with the carboxylate group of Trp [42].

**Figure 13:**
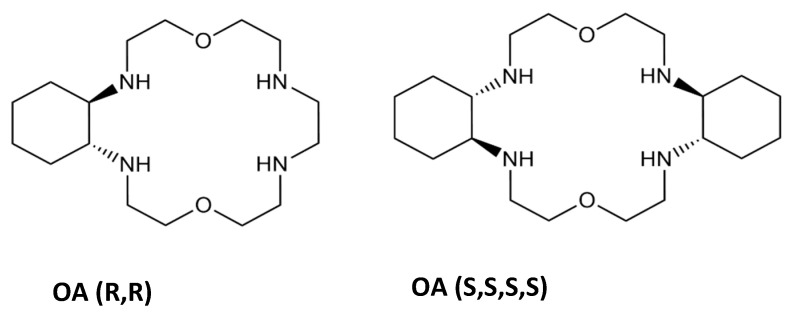
Structure of the oxaazamacrocycle receptors OA (R,R) and OA (S,S,S,S) [53].

Alfonso et al. synthesized two novel oxaazamacrocycles, which presented enantioselective binding with both aspartate and glutamate—OA (R,R) e OA (S,S,S,S) [53]. Only the latter showed considerable selectivity towards the L isomers of these anionic biomolecules, but with relatively low affinities. The former showed higher affinity towards the d-isomer of aspartate. This specificity is given by the positioning of the positive groups and oxygen on the receptor in relation to the analyte that it has affinity towards (Figure 13). This distinct approach in receptor design allowed for the positioning of its charged and polar moieties in proximity to guests’, in a complementary manner. The positive ammonium groups turned towards the cavity of the receptor and the two oxygens, free to form two hydrogen bonds each, facing in the same general direction, is the ideal conformation for the binding of d-Ac-Asp to OA (R,R), allowing the establishment of ionic interactions and one hydrogen bond without the need for many spatial rearrangements of the receptor [53].

**Figure 14:**
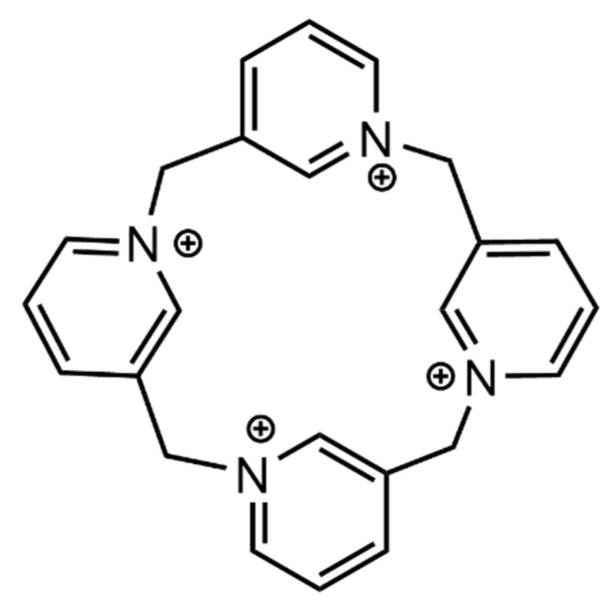
Structure of the receptor calixpyridinium, C4Pyr.

Much like the previous examples, calixpyridinium, C4Pyr is a supramolecular system that has not been target of much attention in biomolecule binding applications (Figure 14). However, this macrocycle presents many similarities to calixarenes, with high solubility and with added cationic groups allowing for the creation of a binding site for small anionic biomolecules. Ren and co-workers tested this receptor for its affinity towards anionic amino acids—it showed to be highly selective for these, when compared to the rest of the proteinogenic amino acids, binding aspartate and glutamate (*K* ≈ 10^3^ M^−1^) solely by ionic interactions [52].

### 3.2. Open-Chain Receptors

Despite the great variety of macrocycles here presented with affinities reaching the μM range, some open-chain receptors can present rigid binding sites with affinities rivalling those of macrocycles [24]. On the other hand, less rigid structures can bring other advantages, e.g., the flexibility to bind larger guests [117].

**Figure 15:**
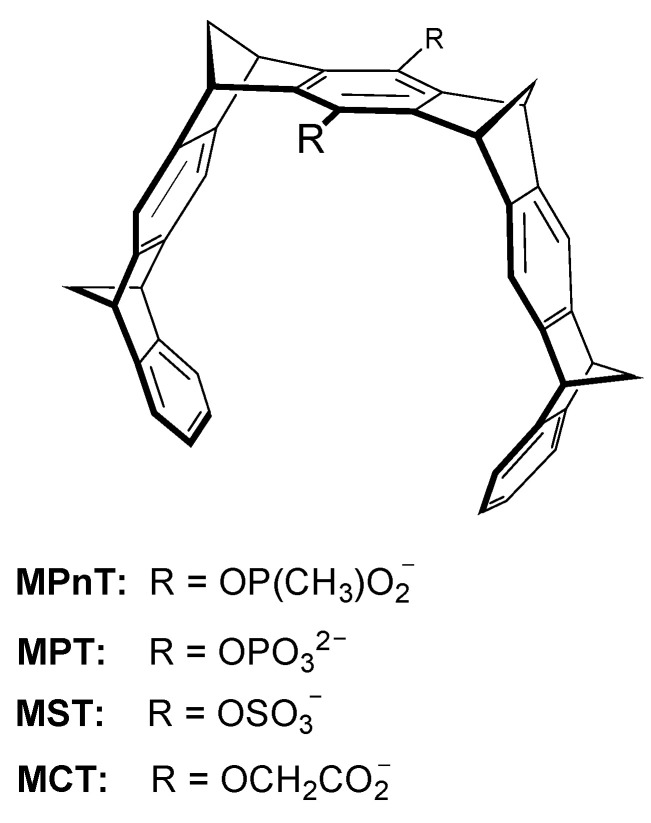
Structure of the Molecular Tweezers reported in Schradder et al. functionalized with phosphonate (MPnT), phosphate (MPT), sulfate (MST), and carboxylate (MCT).

Molecular tweezers are open-chain receptors comprising semi-rigid structures that can bind complementary analytes [24,25,118]. Two studies reported rigid molecular tweezers with the ability to bind Arg and Lys, which were designed with nine adjoining six-membered rings and a hydroquinone group at the center. The two hydroxyl groups in the hydroquinone were modified to incorporate different anionic moieties—phosphonate, phosphate, sulfate and carboxylate—creating four differently-substituted receptors, phosphonate (MPnT), phosphate (MPT), sulfate (MST) and carboxylate (MCT), respectively (Figure 15) [24,25]. These receptors showed different affinities towards Arg and Lys, as well as the acylated and O-methylated modified residues, as shown in Table 1.

**Figure 16:**
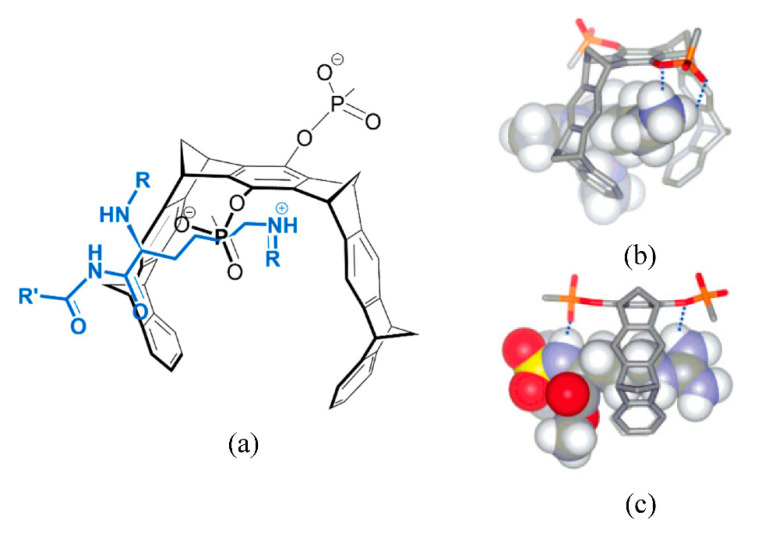
(**a**) Proposed structure adapted from Fokkens et al. and Monte Carlo simulations of the complexes between MPnT and Ac-Lys-OMe (**b**) and Ts-Arg-OEt (**c**)—MacroModel 7.1, Amber*, water, 5000 steps. Reprinted from [24] with permission. Copyright (2005) American Chemical Society.

The first of these receptors to be tested was the phosphonate tweezer, which showed selectivity towards Lys, with even higher affinity towards its α-N/C-Protected form. NMR and ITC studies elucidated the interactions formed in these complexes where all the analytes tested showed a full incorporation of their side chain near the hydroquinone group (Figure 16). The binding process is aided by cation–π interactions between the positively charged groups of the amino acids and the electron-rich interior region of the tweezer, as well as by hydrophobic interactions between the alkyl chains of the analytes and the aromatic groups. However, the formation of the complexes are mainly driven by ionic interactions between the receptor’s anionic groups and the positive charges in the analyte—explaining the higher affinity towards Lys, seeing as arginine presents a more delocalized charge, diminishing the stability of this latter complex [24].

This was supported by later studies with molecular tweezers comprising different anionic moieties, in which the phosphate and sulfonate containing tweezers present the highest affinities, in contrast to phosphonate and carboxylate which present lower charge density. The lower affinity of most of these tweezers to non-protected forms of the amino acids is most likely due to the presence of repulsive forces between the receptor and the carboxylate group of the amino acids [25].

**Figure 17:**
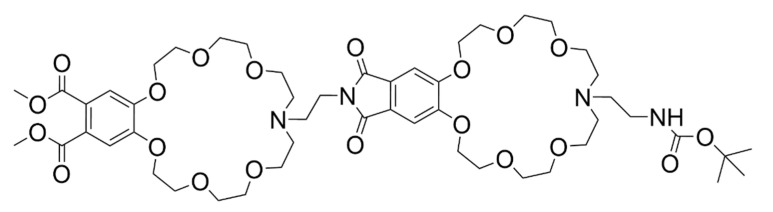
Structure of the molecular tweezer, CEAT, developed by Mandl and König [118].

Another application of this concept was developed by Mandl and König, in which they made a receptor with two modified crown ether moieties, CEAT [118] (Figure 17). These are capable of binding O-methylated Lys, with an affinity of 4.0 × 10^4^ M^−1^, namely by the formation of H-bonds between the crown ether ring and the amines in the amino acid. However, these receptors have rather small affinities when in aqueous buffered solutions, while also needing the presence of a small percentage of methanol, which indicates that they are less suitable for physiological applications [118].

**Figure 18:**
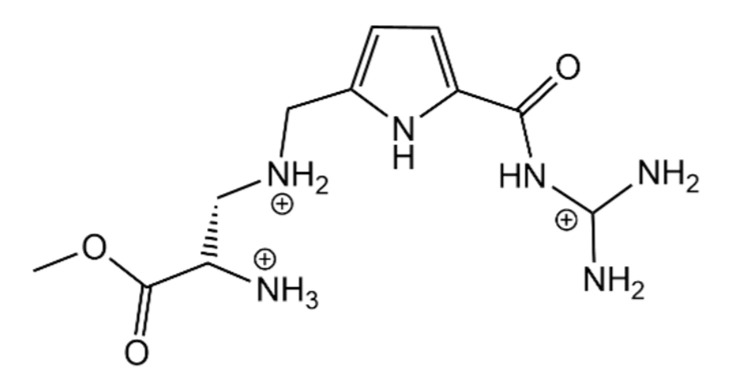
Structure of the guanidinium pyrrole receptor, GP3cat [59].

Schmuck and Geiger also developed an open chain receptor for amino acids, based on a guanidinium pyrrole unit, with 2 added cationic groups, GP3cat (Figure 18). This receptor showed a remarkable increase in affinity towards acetylated l-Ala, in comparison to other mono or di-cationic forms of the receptor. This 1:1 complex is mainly stabilized by ionic interactions between the receptor and the carboxylate of the analyte, as well as by multiple hydrogen bonds that are formed at the same time with the same groups. Despite the extra carboxylate of glutamate, the 1:1 complex is more stable with alanine, although two receptors can bind to 1 molecule of glutamate, which increases the affinity towards this amino acid. Although promising, this system was not studied solely in water, still needing 10% DMSO in order to increase its solubility [59].

## 4. Recognition of Peptides: Taking Inspiration in Nature

One of the main targets for protein detection is the recognition of key elements, most often on the surface of the protein, seeing as this is the region most easily available for binding. For small peptides most of their residues are easily available for binding and is also easier to select a target region for detection. In either case, the best way to achieve this is by the recognition of the peptide side-chains or C and N-terminal amino acid residues [7]. In this section, the focus will be on the previously mentioned receptors, and other, more complex structures, applied in the selective recognition of target amino acid sequences in peptides [22,24,25,26,35,52,121,122,123].

### 4.1. Macrocyclic Receptors

From the receptors presented before, there are a few that have received a larger focus and that their affinity towards certain residues and groups is well defined. Cyclodextrins, for example, have been shown to have affinity for hydrophobic amino acids like phenylalanine, but still bind weakly to the peptides studied in most works, while cucurbit[n]urils, which are selective for hydrophobic and basic amino acids form complexes with peptides and proteins that are significantly more stable, reaching nanomolar affinities in specific cases [37,108,124,125]. Charged macrocyclic receptors, as can be expected, bind oppositely charged residues, being this, together with the hydrophobic effect, the main driving forces for peptide binding in aqueous solutions while other non-covalent interactions may contribute in a small extent for overall binding energy [7,9,33]. However, it must be stressed that these secondary interactions may play a decisive role in the overall selectivity and structure of the complexes.

The simplest and non-modified forms of these macrocycles can be used for selective binding of peptides, which is reported in many studies [21,35,41]. Using these as a starting point, many other receptors have been developed, modifying the original structures to suit the type of amino acids and peptides that are being targeted [40,52]. There are also other types of macrocycles that, while less used for biological applications, have been adapted and show promise in the binding and detection of peptides, as was touched upon in the previous chapters [51,94,95].

#### 4.1.1. Calixarenes

*p*-Sulfonatocalix[4]arene (SC4) binds not only cationic amino acids but also small cationic peptides. Complexes with polycationic peptides are, in general, more stable due to the formation multivalent ionic interactions. One of the first studies with polycationic peptides and SC4 was reported by Morel-Desrosiers and co-workers [22], in which they observe some changes in the way that peptides bind to this receptor, in comparison to the single amino acid. For Lys-Lys, Arg-Arg and Lys-Arg, the *N*-terminal amino acid is enclosed within the cavity of the receptor, with the C-terminal amino acid staying only near the entrance of the macrocycle. NMR studies showed that, between Lys-Lys and Arg-Arg, the latter binds deeper in the SC4 cavity, most likely due to the stabilization of the complex by cation–π interactions. The two other peptides differ slightly in the mode of binding—while Lys-Arg seems to bind with the *N*-terminal amino acid, lysine, in the cavity and arginine remaining outside the macrocycle, the Arg-Lys peptide binds with the C-terminal deeper in the cavity.

The tripeptides tested in this study showed higher stabilization than the remainder of the analytes. This is most likely due to two main factors: the multivalent ionic interactions, due to the presence of an added cationic group, increasing the sites of interaction and, so, increasing the stability of the complex [126]; a greater distance between the C-terminal and the sulfonate groups of the receptor diminishes the electrostatic repulsion between these two negatively charged groups and allows for more stable binding with the *N*-terminal amino acid, deeper in the cavity, and the middle amino acid, at the entrance of the cavity. This study also showed that, in general, the complexes formed with the amino acid l-Arg and Arginine peptides are slightly more stable than their l-Lys counterparts, due to the presence of the guanidinium moiety that may undergo π–π interactions with the calixarene cavity, as explained in Section 3.1.1. [22].

Ghale et al. [127] also used *p*-sulfonatocalix[4]arene for the detection of arginine-rich peptides, using an indicator displacement method. In this study, this receptor was applied for the detection of several cationic analytes in liposomes (with more focus on protamine, a natural occurring peptide with 21 arginine residues) and to study their transport across membranes, mediated by the membrane protein OmpF. The association constants presented in Table 2 further establish that, in general, this macrocycle forms more stable complexes with peptides with higher number of positively charged residues due multivalent ionic interactions, as commented above. Later studies show the same effect with an amphiphilic SC4 and its complexation with heptaarginine and other small arginine-rich cell-penetrating peptides [121].

The high affinities towards multivalent cationic peptides make this receptor a promising one, for in vitro study of several types of peptides—antimicrobial peptides [33], protamines [128], and mitochondrial signaling peptides [129]—but also make it a viable tool for applications in sensing of proteins and their modulation. Selected examples of this include the use of SC4 as a promoter of protein crystallization, protein assembly and as a masking agent, to alter proteins’ surface properties [130,131,132,133,134,135].

**Figure 19:**
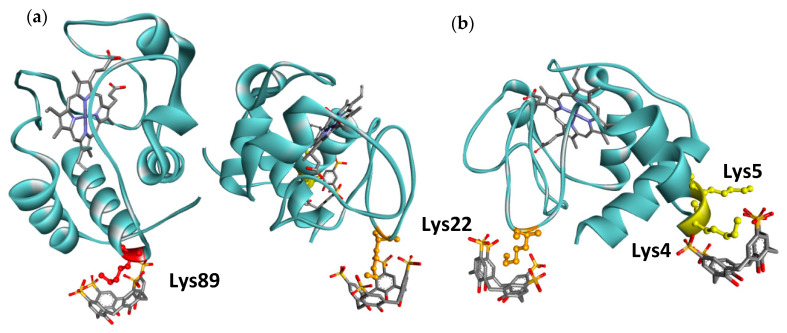
Image of 3TYI (structure obtained by McGovern et al. [131]) created with the software Discovery Studio 2020 Client (BIOVIA, Dessault Systèmes, San Diego: Dessault Systèmes, 2020) (**a**) Asymmetric unit of the crystal structure of the cytochrome c-SC4 complex, showing three interact Table 89. (red), Lys22 (orange), and Lys4 (yellow), at the surface of the protein, with respective interactions in bright orange. (**b**) Side view of one of the proteins in the asymmetric unit for better visualization of the Lys4 binding site; Lys5 is also represented in yellow, for it could form complementary interactions with the SC4 molecule in its proximity.

This is the case of studies with the protein cytochrome c, with characteristic positively charged regions at the surface, composed of several lysine residues, which have a very important role in the protein–protein interactions that Cyt c can establish. McGovern et al. have shown that SC4 binds this protein at three different lysine residues (Figure 19), forming ionic and cation–π interactions with the residue, but being further stabilized by hydrogen bonds with the nearest polar residues and groups, as well as by the hydrophobic effect. The binding of SC4 results in a change in the overall charge at these sites, concealing the lysine residues and altering the protein–protein recognition properties of these biomolecules. Moreover, the changes at the protein surface enhanced the protein’s ability to form crystals, indicating possible uses of SC4, not only in physiological conditions, for function modulation purposes, but also as an enhancer of crystallization [131].

#### 4.1.2. Calixpyridinium

This macrocycle was tested not only with amino acids, as previously mentioned, but with a glutamate dipeptide, to evaluate if the high affinity was maintained or if there were any changes in the binding mode. The same study showed that the calixpyridinium-peptide complex presented very similar stability as the one with only glutamate, which might indicate that the binding occurs mostly in the same way (Table 2) [52]. In the case of the dipeptide it is possible that the *N*-terminal residue is also inside the macrocycle’s cavity but that the electrostatic repulsion between the positive *N*-terminal hinders the binding, resulting in equal affinity to that of l-Glu.

Despite the possible use of this system in the detection of peptides, it remains that calixpyridinium presents higher affinity constants towards nucleosides, in particular towards ATP, and so, being more interesting as a receptor for this anionic molecule [136].

#### 4.1.3. Pillararenes

Much like calixpyridinium, pillararenes previously mentioned either were not further tested for their affinity towards peptides or were only tested with a couple of small peptides, yielding very similar results as for the binding of the respective amino acids. Carboxylatopillar[5]arene (CP5) was tested for two peptides—Ala-Arg-Ala and Ala-Lys-Ala—in the same study mentioned before [26]. NMR studies showed that, in this case, the CP5 receptor only encapsulated the side chain of the middle basic amino acids, stabilizing both by ionic interactions and the hydrophobic effect. Similar to the amino acid results, this macrocycle has five times higher affinity for the peptide with Arginine, most likely due to the formation of cation–π interactions, giving it a rather high selectivity for the presence of this amino acid.

#### 4.1.4. Cucurbiturils

The binding of peptides to CB[n] constitutes a remarkable success story in the field of host-guest systems comprising this type of biological molecules. As previously mentioned, CB[n] generally display higher affinity for hydrophobic aromatic amino acids being the release of high energy water molecules from the macrocycle’s cavity the main thermodynamic driving force contributing for the stability of the complexes [105]. Ion-dipole and hydrogen bonding interactions between carbonyls portals of the receptor and specific functional groups present in the peptide are believed to contribute in a less extension for the overall driving force but may have a decisive role in the observed selectivity. The high affinity interactions of *N*-terminal aromatic residues, in particular phenylalanine, with CB7 and CB8 constitutes a paradigmatic target for the selective binding behavior of this class of hosts [7,37,108,122,124]. For example, Phe-Gly and CB7 form a 1:1 complex with a *K* = 3.0 × 10^7^ M^−1^ while the complex formed with Gly-Phe is much weaker (*K* = 1.3 × 10^3^ M^−1^), as is summarized in Table 2 [122]. In both cases the phenylalanine residue is included in the CB7 cavity. However, while in Phe-Gly this binding mode favors attractive ion-dipole interactions between the carbonyl portals and the ammonium group and minimizes the repulsion of the portal with the carboxylate group, for Gly-Phe the carboxylate is closer to the carbonyl group precluding optimal inclusion by electrostatic repulsion. Furthermore, the attractive interaction between the ammonium group in the Gly residue with the carbonyl portals of the receptor imposes a significant entropic penalty due to conformational restriction of the guest [122]. Although displaying slightly lower binding constant, both tryptophan- and tyrosine-glycine dipeptides display similar behavior to that described for Phe-Gly/Gly-Phe [122]. It is also worth noting that the smaller CB6, in contrast to CB7 and CB8 (see below), does not significantly bind peptides with terminal aromatic residues, showing much higher affinity for those comprising terminal lysine [122].

**Figure 20:**
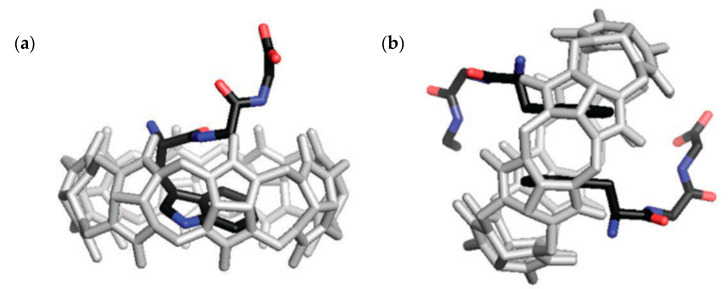
Crystal structure of the complexes with CB8 (structure in white) and the peptides (structures in black)—(**a**) CB8-(Trp-Gly-Gly) and (**b**) CB8-(Phe-Gly-Gly)2. Despite the formation of 1:2 complexes for both these peptides, Heitmann et al. were only able to obtain this complex structure for Phe-Gly-Gly. Reprinted with permission from [108]. Copyright (2006) American Chemical Society.

Urbach and co-workers [37,108] focus their earlier work on the binding of *N*-terminal amino acids to the larger CB8. As described above for CB7, CB8 also binds preferentially peptides containing aromatic amino acids at the *N*-terminal. These receptor-analyte pairs can form 1:1:1 complexes in the presence of methyl viologen as an auxiliary guest [37], but can also assist the dimerization *N*-terminal aromatic peptides in the absence of methyl viologen by forming 1:2 host–guest complexes. CB8 shows selectivity towards Trp-Gly-Gly and Phe-Gly-Gly (Figure 20) [108], forming CB8-peptide_2_ complexes with overall binding constant up to 3 × 10^11^ M^−2^ (Table 2) [108,137,138]. While the arguments described above to rationalize the selective binding of *N*-terminal aromatic residues to CB7 are also likely to hold here, the crystal structures shown in Figure 20, besides showing those interactions, also demonstrate that hydrogen bonding between the amide protons and the oxygen atoms in the carbonyl portals may also contribute to increase the stability of these complexes [108].

**Figure 21:**
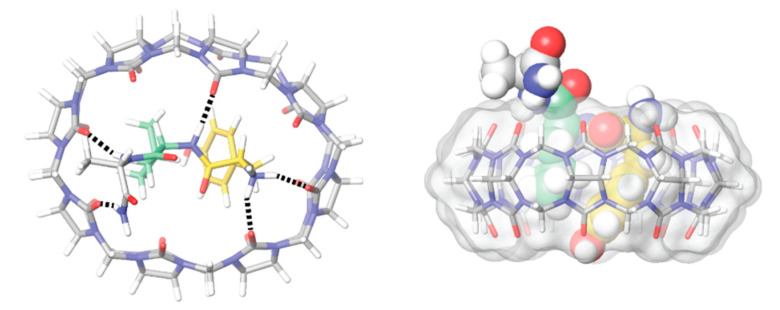
Semiempirical structure obtained by Smith et al. (2015) of the complex CB8-(Tyr-Leu-Ala)—up view (left) with the electrostatic interactions represented by dashed lines, and side view (right), where it is observable the two *N*-terminal residues inserted in the cavity of the macrocycle. Reprinted with permission from [125]. Copyright (2015) American Chemical Society.

More studies have been done with CB8, screening for any specificity among peptides with *N*-terminal aromatic residues [125]. Most of these were performed with tripeptides, in which the *N*-terminal residue was aromatic, and the two others varied, in order to assess their importance in the binding process. It was discovered that, even though most of the peptides bind in a 2:1 ratio to CB8, the sequence Tyr-Leu-Ala showed high affinity (in the nM range) for this macrocycle. However, it bonded in a 1:1 ratio instead of the 2:1 observed previously. This was not only true for Tyr-Leu-Ala; when tyrosine was the *N*-terminal amino acid, some peptides formed a 1:1 complex with CB8, because the adjacent amino acid could also be encapsulated in the CB8′s cavity along with l-Tyr, leaving the C-terminal at the entrance of the macrocycle (Figure 21). These peptides were of the Tyr-X-Ala kind (with X being either Leu, Lys, Phe or Tyr), and did not bind in this way to CB8 when X and Ala were switched. This high affinity towards a Tyr-Leu-Ala peptide is a step towards developing simple supramolecular systems that mimic the specificity displayed by antibody-based receptors.

The same researchers proceeded to do further screening, namely with a library of 144 tripeptides, X1-X2-Ala. X1 consisted of amino acids with large hydrophobic or cationic side chains, seeing as this will be the residue farther inside CB8s cavity and the larger size is needed in order to have 1:1 binding. X2 corresponds to 18 possible amino acids, leaving off only Trp and Cis from the existing 20 human physiological amino acids, due to possible interference in the measurements [123].

This study showed that CB8 had affinity towards peptides with Tyr, Phe, Ile, and Leu at X1 and Tyr, Phe, Leu, and Lys at X2. Sequences with Met, Arg and Lys also showed considerable affinity. The affinity towards methionine at the X1 position was the one explored in this study, seeing as Met is often found on *N*-terminal of proteins before further post-translational modification, being of particular interest to study these PTM mechanisms [139].

Analyzing the binding by ITC studies, showed that the second amino acid should be large and hydrophobic or cationic, but without branching in the beta carbon, while the third amino acid should be small, so as to avoid having stereochemical hindrances [123]. These types of tripeptides show sub micromolar affinities towards CB8 and are also a plausible target for the detection of proteins by their *N*-terminal, seeing as, despite methionine being a common site of excision, large amino acids in the position next to Met can block the binding of the enzymes that catalyze this process, which distinguishes these *N*-terminal sequences from the excised ones [139]. Additionally, this functionality of the macrocycle can be used as a protection against excision of the *N*-terminal, for proteins presenting these sequences [109].

More recent studies focus on different modes of peptide binding to the receptor CB8, with a focus on peptides containing the pairs of residues Tyr-Leu or Phe-Leu. Here it is shown that CB8 can bind to these residues even if farther away from the *N*-terminal, with a diminished affinity by one order of magnitude, and that the binding can occur not only in a 1:1 ratio, but also in a 1:2 ratio for longer peptides with repeated Tyr-Leu sequences (Tyr-Leu-Ala-Gly-Gly-Ala-Leu-Tyr) [140].

**Figure 22:**
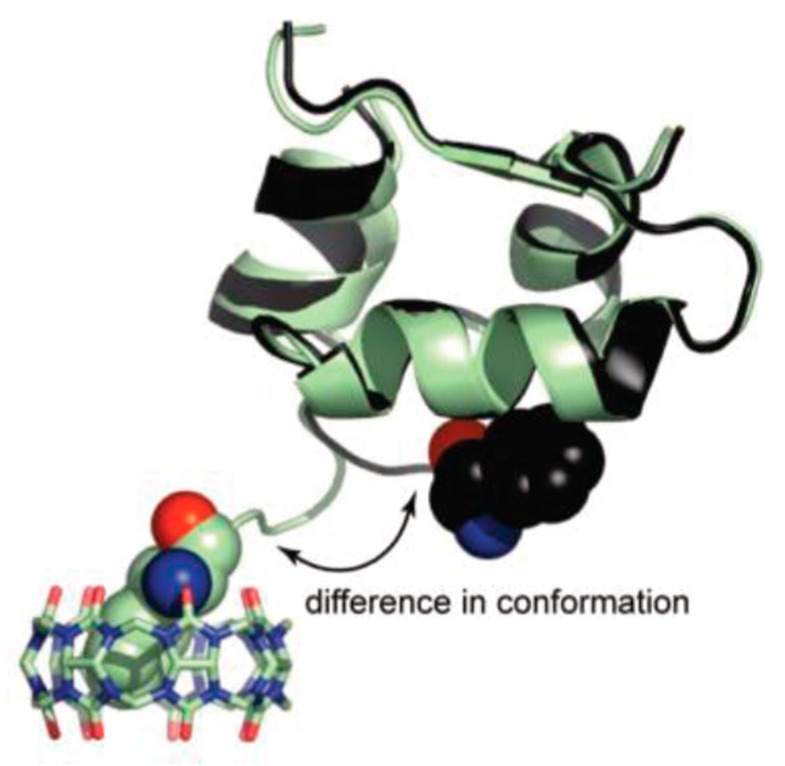
Crystal structure of the complex formed between CB7 and human insulin (light green) and of free insulin (black). Reprinted with permission from [124]. Copyright (2011) American Chemical Society.

Due to the presence of aromatic amino acids in insulin’s *N*-terminal, this protein comes up as a possible target for this type of receptors. With this in mind, Chinai et al. evaluated the affinity of this system towards human insulin and a mutant variety without the *N*-terminal aromatic amino acid [124]. This latter variant showed an obvious decrease in affinity towards CB7 when compared to human insulin. The latter showed similar affinity towards the macrocycle as Phe-Gly-Gly. Furthermore, it was observable a slight change in conformation in insulin to allow for this binding, exposing the *N*-terminal to the complexation, but without altering the overall structure of the protein. This implies that even less exposed *N*-terminal amino acids could be targets for this type of sensing without any major interference with the rest of the structure, while also distinguishing them from other aromatic amino acids in a protein’s surface or in a peptide’s sequence (Figure 22). Additionally, the *N*-terminal is usually a more exposed and flexible region in proteins, being possible that this could be more easily transposed to studies with peptides with a corresponding sequence.

As can be seen by this example, CB[n]’s can be used for the detection of proteins, due to its specificity towards aromatic and cationic residues. Control over protein assembly and function is another route that is being explored for this supramolecular system [9,141,142,143,144,145,146,147,148]. Brunsveld and co-workers have used CB8 as a tool to aid in the dimerization of proteins, making use of the target Phe-Gly-Gly motif as an *N*-terminal modification, in order to better control enzyme activity [149] and protein assemblies [142].

The second example consists of the use of a modified peptide, with a 14-3-3 protein binding site at the C-terminal and with an Phe-Gly-Gly moiety at the *N*-terminal. This structure allows for the binding of this peptide to the 14-3-3 protein, which presents two binding sites, with affinities comparable to those of the physiological targets. CB8 can be added to stabilize this bivalent complex by 14 times, by complexation of the two *N*-terminals of the peptides bound to this protein, serving as a tool to switch the “availability” of the 14-3-3 binding site [142].

A more recent study reported the role of CB8 as a modulator of enzymatic activity using caspase 8, an enzyme that is typically only active upon dimerization. In this work Dang et al., after observing that the Casp-8 *N*-terminal residues of the two monomers are in close proximity in the dimeric active state, engineered this region of the Casp-8 to have an Phe-Gly-Gly motif [149]. This motif allowed for the use of CB8 to control the dimerization in the inactive mutants and resulted in an easily modulated, dimeric form of Casp-8 active towards a synthetic ligand as well as its physiological substrate, caspase-3.

The CB[n]’s affinity towards basic residues has also been explored in the scope of protein recognition. Namely, Crowley and co-workers tested CB7′s affinity towards a demethylated lysine in a protein, *Ralstonia solanacearum* lectin (RSL). Despite having other methylated lysine residues in its sequence, CB7 recognized selectively the Lys34Me_2_, which is located in a more accessible region of RSL, yielding an affinity in the mM range (Table 2). Moreover, CB7 has also been successfully exploited in this context to create novel protein assemblies around CB7 trimers and tetramers, in the crystal structure of the CB7-RSL complexes [150].

#### 4.1.5. Cyclodextrins

As described in the previous sections, the properties of these natural occurring macrocycles translate to higher affinities towards aromatic residues or residues with small, apolar side chains in the context of peptides and proteins [35,41,119,151,152]. The same types of interactions occur with both amino acids and peptides—the binding at the cyclodextrin cavity is generally driven by hydrophobic effects, with some hydrogen bonds formed with the outer shell of the macrocycle. In most cases, these interactions only allow for small affinities, only surpassing mM affinities in 50% methanol solutions [152], or with further modification of the original macrocycle [151].

#### 4.1.6. Crown Ethers

**Figure 23:**
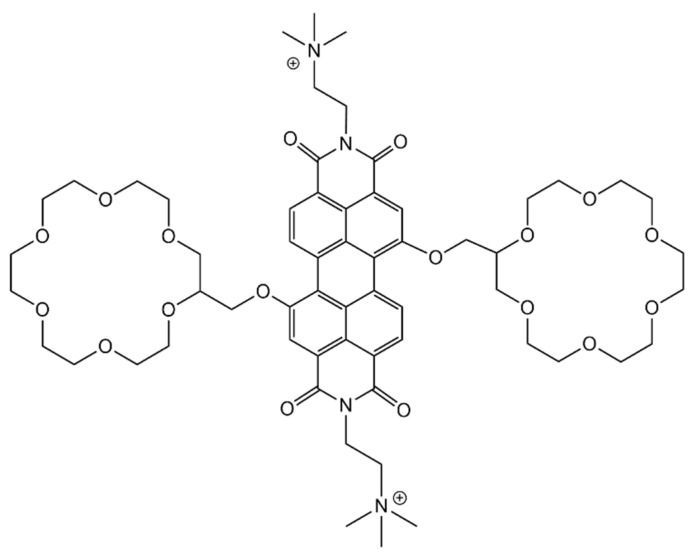
Structure of the crown ether molecular tweezer, perylene bisimide dye (PBI) [153].

A different approach for detection of aromatic amino acids and small peptides containing these residues was presented by Weißenstein et al. [153]. In their studies they joined the properties of two different moieties—as readout mechanism they used a perylene bisimide dye (PBI), which has high fluorescence quantum yield and can be quenched by photoinduced electron transfer (PET)—this process can occur when this group forms bonds with electron rich moieties, e.g., aromatic amino acids which can bind to PBI by π–π stacking of aromatic groups; the other distinct moiety is composed of two crown ethers that serve as a receptor for ammonium cations, e.g., the *N*-terminal of short peptides or amine groups present in cationic amino acids. The presence of two crown ethers allows for the binding of two analytes, while PBI can form two π–π stacking bonds with the aromatic amino acids (Figure 23).

The highest affinity of PBI, at least one order of magnitude above that of other peptides and amino acids tested, was towards Ala-Trp. This peptide is believed to have the optimal characteristics for binding to occur due to the separation between the ammonium ion at the *N*-terminal and the aromatic residue. It must be ideal for the formation of interactions between the *N*-terminal ammonium group and the crown ether and between the aromatic side chain and the perylene bisimide unit, at the same time. The downside of this probe is that its studies were not made in water (probably due to poor solubility of the receptor) and so, the conclusions taken, and the overall system are, most likely, not applicable in water and physiological conditions.

**Figure 24:**
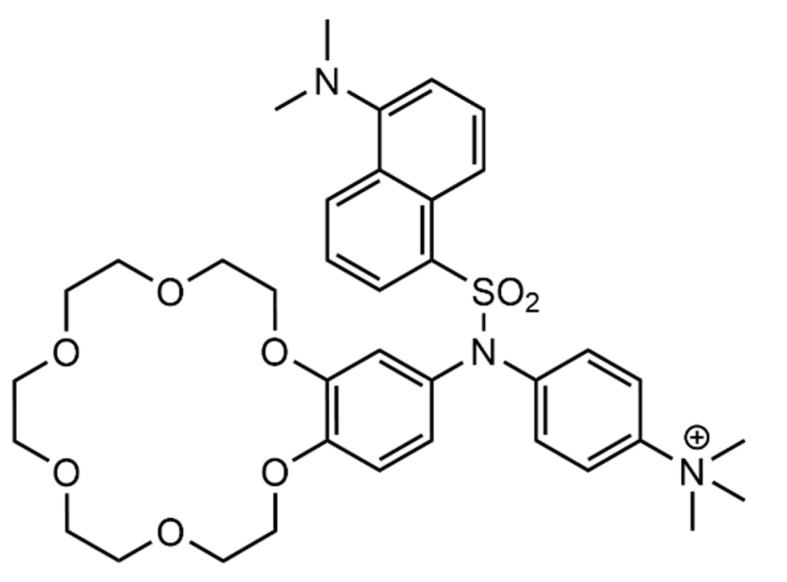
Structure of the crown ether and trimethylammonium sensor, CENMe_3_ [154].

Similar systems have been explored before, in water, as is seen as early as 1998 in the work of Hossain and Schneider [154]. They used a crown ether and a trimethylammonium moieties to bind to the peptide’s *N* and C-terminals, respectively. These moieties were connected by a hydrophobic linker, with an added fluorescent dansyl unit for the detection of the biomolecules (Figure 24). The synthesized receptor, CENMe_3_, reached affinities up to 10^3^ M^−1^, for both Gly-Phe-Gly and Gly-Trp-Gly, at least 1 order of magnitude above the affinities towards other tested peptides. Even though this receptor shows specificity towards the middle positioning of an aromatic residue in this type of peptides, for biological applications, a 10^3^ M^−1^ affinity constant is not the most desired value, since physiological concentrations are much lower than those needed for considerable complex formation.

#### 4.1.7. Cavitands

**Figure 25:**
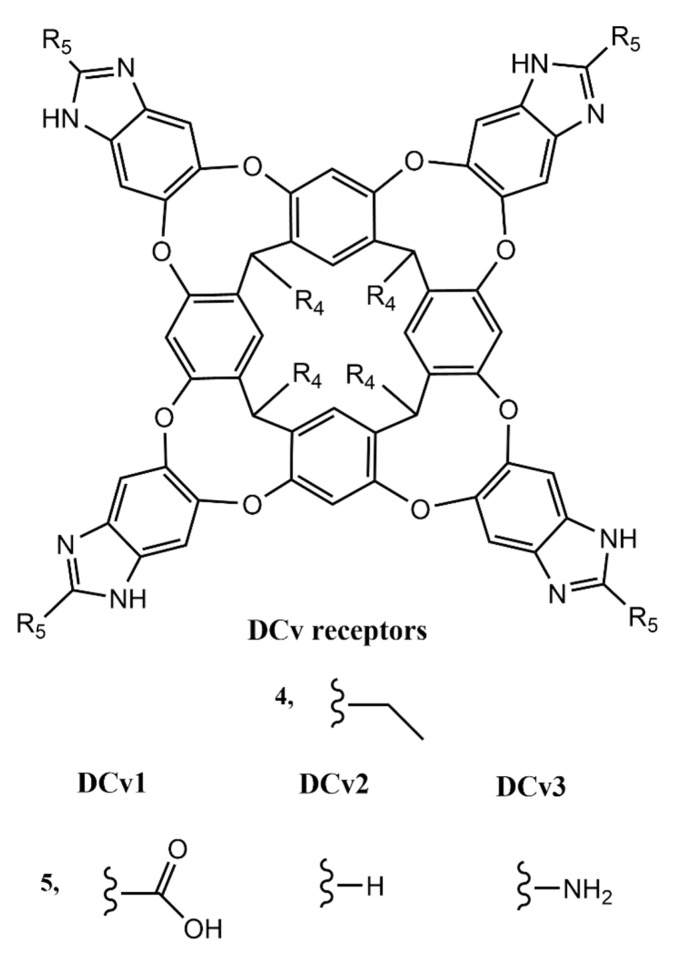
Structure of the deep cavitands, DCv1, DCv2 and DCv3 [158,159].

Cavitand receptors have seen multiple applications in recognition of small organic molecules, including peptides and proteins. Some examples comprise the integration of these macrocycles in sensing scaffolds [155,156], nanostructures [157], as well as their use in solution [158,159,160]. The latter includes a group of self-folding deep cavitands, DCv (Figure 25), which have been applied in the detection of select histone sequences and their respective post-translational modifications (PTM) in water. These interact with lysine residues, showing different affinities depending on the R_5_ group’s nature (Figure 25) and the degree of methylation of the guest. The complexes established are stabilized mainly by cation–π interactions but also with the contribution of a variety of other electrostatic interactions [157,159,160]. Furthermore, all three of the deep cavitands schematized have been applied in a dye displacement assay for the screening and monitoring of the PTM on a select library of peptides. These included several methylation sites that are recognized by both histone methyltransferase and demethylase, allowing for an analysis of their activity at several of the specific sites in which they act upon [159].

### 4.2. Open-Chain Receptors

Besides the obvious interest in peptide and protein sensing, many receptors for peptides have been explored focusing on the role of these biomolecules as targets for the action of several pharmacological agents. The antibiotic vancomycin and its derivatives are amongst the most recognized examples.

**Figure 26:**
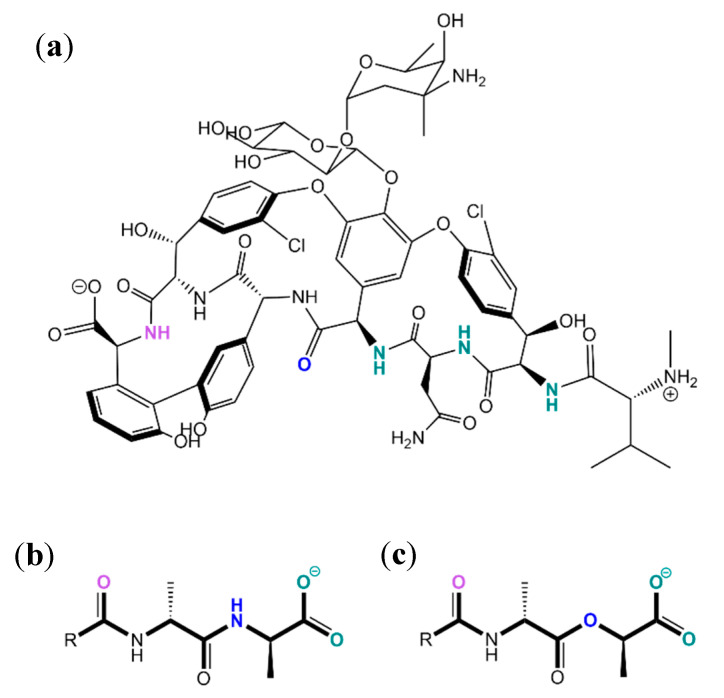
(**a**) Vancomycin, (**b**) its target sequence in the cell wall of gram positive bacteria, d-Ala-d-Ala-OH and (**c**) the mutated sequence, d-Ala-d-Lac-OH. The sites in vancomycin highlighted in pink, blue and green interact with the same colored groups in the sequences. In the mutated sequence, one of the hydrogen bonds is broken and there is instead electrostatic repulsion between the groups in blue [6].

Vancomycin binds a specific sequence in the cell wall of gram-positive bacteria, d-Ala-d-Ala-OH, disabling the action of a transpeptidase at this site, which has a very important role in the crosslinking of several peptide strands in the peptidoglycan layer and stabilizing it. Without this crosslinking, the cell envelope is severely destabilized, and lysis of the cell will occur if there are variations in the local osmotic pressure. In drug resistant strands of the same bacteria, this target sequence is mutated to d-Ala-d-Lac-OH. This change in one amino acid breaks a bond between vancomycin and the peptide and adds electrostatic repulsion between the two oxygens indicated below (Figure 26). This diminishes the affinity constant of vancomycin by two orders of magnitude, but does not affect the binding of the bacterial transpeptidase [6].

**Figure 27:**
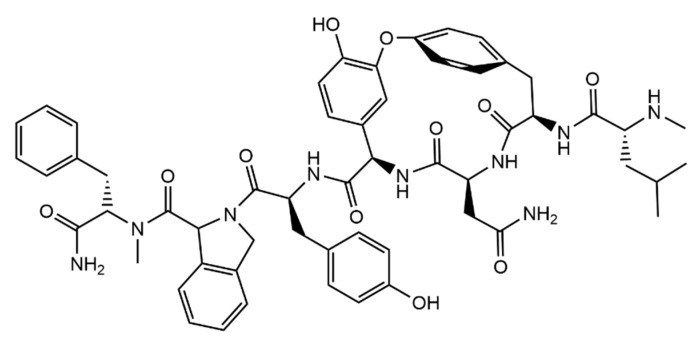
Structure of the peptidyl vancomycin derivative receptor, VD1 [161].

In order to circumvent bacterial mechanisms of resistance, Ellman and co-workers [161] synthesized a library of water soluble receptors, based on vancomycin’s structure, but with a peptidyl sequence in the region where the repulsive forces were introduced when a mutation occurred. The peptidyl group has freer rotation than the rigid group of vancomycin, and this will allow for the increase of the distance between receptor and peptide, diminishing the effect of the repulsion when binding occurs. The receptor shows better complementarity to the sequences and, so, the highest affinity was observed for VD1 (10^4^ M^−1^), represented in Figure 27.

In more recent studies, with other receptors, smaller libraries are being utilized, taking advantage of information obtained from previous studies to focus the design of new receptors towards the properties that are needed [162].

In a study by Schmuck and Heil, with the tetrapeptide *N*-Ac-d-Glu-l-Lys-d-Ala-d-Ala-OH—EKAA—in mind as a target (a bigger fragment of the peptide recognized by vancomycin), a small library of water-soluble receptors was created, with 512 members. They used a similar strategy as the one used for VD1, synthesizing these receptors with a peptidyl chain, with three variable residues, adding the guanidiniocarbonyl pyrrole binding motif (GCP), which was previously shown to bind to the C-terminal of peptides (as mentioned in Section 2.3, relative to the binding of single amino acids) [117]. The GCP binding motif shows similarities with vancomycin in the types of interactions established with the carbonyl group of this sequence with several H-bonds stabilizing both complexes. In the GCP-peptide complex there is the additional ionic bond previously mentioned.

**Figure 28:**
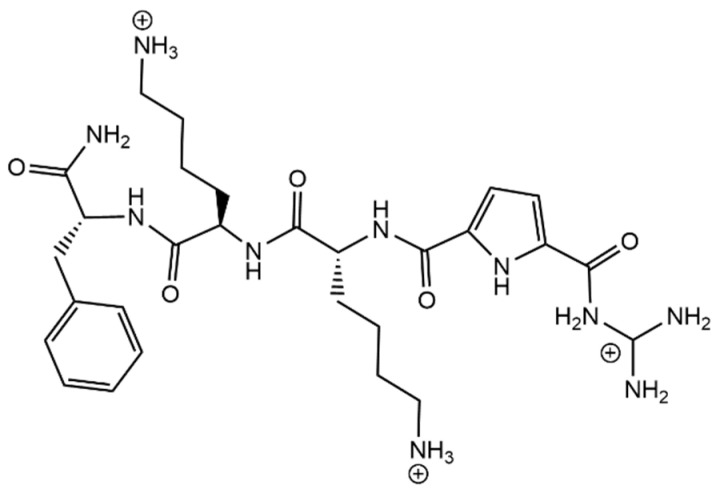
Structure of the peptidyl Guanidiniocarbonyl pyrrole receptor, GCP-KKF [163].

The residues of the peptidyl group varied between Lys, Tyr, Ser, Glu, Phe, Val, Leu, and Trp, being expected that some of these can form more ionic interactions with the analyte and further stabilize the resulting complex. Due to the pyrrole moiety it was possible to monitor the binding of the target peptides with the receptors from the variations in the absorption and fluorescence spectra of the receptors. It was found that the receptor with the sequence Lys-Lys-Phe, GCP-KKF (Figure 28), was the one which presented higher affinity towards both EKAA and the opposite sequence, AAKE, but with markedly higher affinity towards the peptide with Ala at the C-terminal (K_a_ = 1.71 × 10^4^ M^−1^, 4 times higher than that for AAKE), indicating the importance of the C-terminal in the binding of the peptide. Furthermore, the lysine residue also takes part in the binding with the C-terminal, stabilizing the binding to the tetrapeptide further [163].

The receptor which showed highest affinity towards EKAA was also tested for its affinity towards other tetrapeptides, with variable residues, originating a library of ~300 target tetrapeptides, including those with d-Ala and d-Lac in the C-terminal (the first similar to the native sequence of Gram positive bacteria peptidoglycan peptidyl chain and respective mutated sequence, related to vancomycin resistance). The GCP-KKF receptor showed an even higher affinity towards Ac-d-Glu-d-Glu-d-Glu-d-Glu-OH (*K* = 2.65 × 10^4^ M^−1^) and a very accentuated stereoselectivity for d-Ala in d-Glu-d-Glu-d/l-Ala-d-Glu-OH peptide (almost 4× higher than for l-Ala, *K*_a_ (l stereoisomer) = 1.40 × 10^3^ M^−1^ and *K*_a_ (d stereoisomer) = 4.50 × 10^3^ M^−1^) [164].

This shows that focusing on libraries and taking inspiration on known and well-studied host-guest complexes helps to understand the bonds created and how to further stabilize other peptide-receptor complexes [6,163,164].

#### Molecular Tweezers

Molecular tweezer and clips constitute another example of small supramolecular receptors that have been widely explored for the recognition of peptides. These compounds offer an (often electron-rich aromatic) pocket-like binding site, which is usually decorated with functional groups that may provide additional sites of interaction and water-solubility [165,166,167].

The most popular molecular tweezers with applications in the recognition of amino acids and peptides are probably those developed by the Klärner group, which bind preferentially basic amino acid lysine and arginine, as mentioned in Section 3.2. [167]. Some of these compounds show a tendency to form self-assembled dimers, a process that may compete with the complexation of selected targets and lead to concentration dependent apparent binding constants [168]. However, anionic tweezers such as the ones shown is Figure 15 (see Section 3.2) are fluorescent, allowing the monitorization of the complexation process and determination of the binding constants at low concentrations [168]. Besides the amino acids tested, a small library of bioactive peptides with basic residues was also investigated, with importance and applications in several fields [167]. The results showed that the stability of the complexes depends on the anionic groups decorating the receptor and increases according to the following trend: O-methylenecarboxylate < phosphonate < sulfate < phosphate. Due to their electron-rich cavities and anionic functional groups this type of receptor fails to bind peptides lacking basic amino acids, showing, in the other hand, high affinity (μM range) for peptides bearing arginine and/or lysine residues, being the last generally more stable probably due to charge delocalization in the guanidinium groups. The highest affinities were reported for the peptides comprising several adjacent lysine residues at the *N*-terminal which provide more sites to form ionic interactions while minimizing the repulsion of the C-terminal [25].

In general, the complexes seem to be slightly more stable in water than in the methanol, demonstrating the important role of the hydrophobic effect that, nevertheless, is counterbalanced by the increasing strength of the ionic interactions in the organic solvent. Noteworthy, although the binding constant present modest variation upon changing from aqueous to organic media, NMR studies clearly showed that the type of complexes can be dramatically different depending on the solvent: in water the formation of inclusion complexes predominate while methanol favors external complexes [168]. However, it should be stressed that this behavior cannot be generalized and depends on the nature of the anionic groups.

The phosphonate, phosphate and sulfate containing molecular tweezers all complexed peptides with at least one l-Lys residue, while the carboxylate containing tweezer had rather low affinities or did not bind at all to the peptides studied, similarly to their behavior in the binding of the amino acids.

The phosphate molecular tweezer, MPT, has been of great interest, due to its high affinity and specificity towards the positive residues of peptides. Early on, they have been tested as a possible pharmacological agent [25,169,170,171] and showed positive therapeutic effects in tests with animal models of several diseases, without significant cytotoxicity—they bind to amyloidogenic lysine and arginine-rich proteins (e.g., α-synuclein), inhibiting their aggregation [25]; MPT also has shown the ability to disrupt and distort viral envelopes (e.g., Zika, Ebola [170], HIV [169,170]), due to their high levels of sphingomyelin and cholesterol. The carboxylate molecular tweezer, MCT, also shows to inhibit viral activity and disrupt the viral envelope, despite not binding strongly with cationic residues—it was hypothesized, in a 2020 study [171], that the MCT and MPT tweezers are able to interact at virus-like lipid rafts, forming inclusion complexes with sphingomyelin, and disrupt them at the interface with the rest of the membrane, by increasing the already high surface tension of these envelopes.

Other studies explored the importance of the linker in these tweezer receptors, showing again that it is necessary to have some rigidity in the linker between the “arms of the tweezer” and a certain angle and distance between the arms to be able to encase the peptide between them [172,173,174].

### 4.3. Self-Assembled Coordination Cages

**Figure 29:**
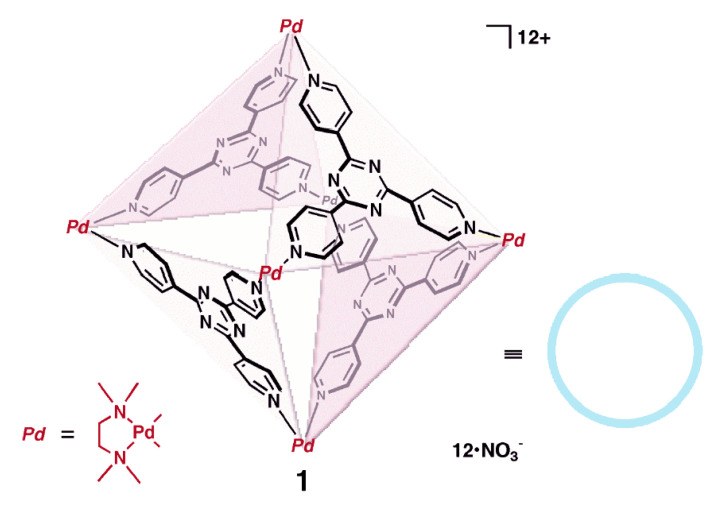
Chemical structure of the Fujita’s Pd6L4 coordination cage. Reprinted with permission from [179]. Copyright (2005) American Chemical Society.

Metal organic capsules (or self-assembled coordination cages) constructed from organic ligands and metal ions often display well defined binding pockets, suitable to accommodate complementary guest molecules in their interior. These have been widely explored as receptors for a variety of molecular targets [175,176,177]. The positively charged Pd_6_L_4_ coordination cage (Figure 29) reported by Fujita was shown to bind complementary oligopeptides with high affinity and impressive sequence selectivity [178,179]. The Pd_6_L_4_ binding pocket was found to be large enough to accommodate up to three amino acid residues in its interior. By analyzing tripeptides with different sequences strong binding was observed for the Ac-Trp-Trp-Ala-NH_2_ sequence (*K* > 10^6^ M^−1^) while very similar sequences, such as Ac- Trp-Ala-Trp-NH_2_, Ac-Ala-Trp-Trp-NH_2_, Ac-Trp-Trp-Gly-NH_2_, Ac-Trp-Tyr-Ala-NH_2_ showed much lower affinity (affinities of the first two sequences can be seen in Table 2) [179]. The results suggest that the cooperative interactions between the two indole rings and the Ala methyl group with the cage and as well intramolecular interactions between the residues (such as π–π and CH-π) may impart a decisive contribution to the thermodynamic driving force. This seems to put in evidence the potential advantages of using receptors with cavities large enough to accommodate more than one residues in its interior as potential strategies to reach exquisite selectivity [179].

More recently, Nitschke and co-workers reported a large Fe_8_L_6_ cubic coordination cage assembled from Zn-porphyrin based ligands and iron(II) metal ions, FeZnP, able to encapsulate guest molecule containing imidazole functional groups that were anticipated to interact with the Zn-porphyrin capsule walls [180]. After demonstrating the affinity of the capsule for this type of guests, the authors investigated the ability of the nanocontainer to bind peptides with histidine residues in a 1:1 acetonitrile:water solvent system. Clavanin A, a peptide antibiotic, containing four histidines among its 23 amino acids was found to bind strongly to the capsule with a dissociation constant of 80 nM in a 1:2 binding manner (Table 2). A less hydrophobic peptide containing only three histidines was found to bind 100-fold more weakly, while peptides lacking histidine residues did not interact at all.

**Table 2:** Binding constants (*K*_a_) for the formation of host-guest complexes between small peptides and synthetic supramolecular receptors.

Receptor	Guest	*K*_a_ (M^−1^)	Conditions	Method	
SC4	Arg-Arg	7.00 × 10^3^	90% water/10% Deuterium oxide Phosphate Buffer 10 mM pH 8, 20 °C	^1^H-NMR	[22]
	Arg-Lys	3.90 × 10^3^			
	Arg-Arg-Arg	3.30 × 10^4^			
	Lys-Lys	3.40 × 10^3^			
	Lys-Arg	3.70 × 10^3^			
	Lys-Lys-Lys	3.30 × 10^4^			
	Trp-Lys-Arg-Thr-Leu-Arg-Arg-Leu	1.20 × 10^6^	HEPES buffer 10 mM pH 7, 20 °C	Fluorescence Spectroscopy	
	Protamine	1.20 × 10^9^	Phosphate buffer 10 mM pH, 25 °C		[127]
	Cyt C binding site 1	1.20 × 10^3^	20 mM Potassium dihydrogenophosphate, 50 mM NaCl, 1 mM sodium ascorbate, 10% D_2_O, pH 6, 30 °C		[131]
	Cyt C binding site 2	6.30 × 10^2^			
	(Arg)_7_	7.00 × 10^7^	HEPES buffer 10 mM pH 7, 25 °C	Fluorescence Spectroscopy	[121]
SC4-C5	(Arg)_7_	2.90 × 10^7^			
	Trp-Lys-Arg-Thr-Leu-Arg-Arg-Leu	2.80 × 10^6^			
C4Pyr	Glu-Glu	1.7 × 10^3^	Water pH 6		[52]
CP5	Ala-Arg-Ala	4.20 × 10^3^	Deuterium oxide pD 7.2, 25 °C	^1^H-NMR	[26]
	Ala-Lys-Ala	7.50 × 10^2^			
CB7	Phe-Gly	3.00 × 10^7^	Water, 25 °C	ITC	[122]
	Gly-Phe	1.30 × 10^3^			
	Tyr-Gly	3.60 × 10^6^			
	Gly-Tyr	2.00 × 10^2^			
	Trp-Gly	5.60 × 10^5^			
	Gly-Trp	2.80 × 10^2^			
	Phe-Gly-Gly	2.80 × 10^6^	Sodium Phosphate 10 mM pH 7, 27 °C		[124]
	Insulin	1.50 × 10^6^			
	RSl-Lys34Me_2_	1.00 × 10^3^	20 mM Potassium Phosphate, 50 mM Sodium Chloride,1.2 mM α-methyl-l-fucoside, 90%H_2_O/10%D_2_O, pH 6, 30 °C	^1^H^15^N HSQC	[150]
CB8	Trp-Gly-Gly	1.30 × 10^5^	Sodium Phosphate 10 mM pH 7, co-binding with MV, 27 °C	ITC	[37]
	Gly-Trp-Gly	2.10 × 10^4^			
	Gly-Gly-Trp	3.1 × 10^3^			
	Gly-Gly-Trp-Gly-Gly	2.50 × 10^4^			
	Phe-Gly-Gly ^a^	1.50 × 10^11^	Sodium Phosphate 10 mM pH 7, CB8-peptide_2_ complex, 27 °C		[108]
	Trp-Gly-Gly ^a^	3.60 × 10^9^			
	Tyr-Leu-Ala	1.39 × 10^8^	Sodium Phosphate 10 mM pH 7, 27 °C		[37]
	Tyr-Lys-Ala	5.00 × 10^6^			
	Tyr-Phe-Ala	3.44 × 10^6^			
	Tyr-Tyr-Ala	1.43 × 10^6^			
	Met-Phe-Ala	7.14 × 10^6^			[123]
	Met-Tyr-Ala	4.00 × 10^6^			
	Met-Leu-Ala	1.39 × 10^6^			
	Met-Lys-Ala	3.85 × 10^5^			
	Met-Tyr-Gly-Gly-Tyr	6.25 × 10^6^			
	Met-Leu-Gly-Gly-Tyr	3.33 × 10^6^			
	Leu-Met-Gly-Gly-Tyr	6.25 × 10^6^			
	Met-Lys-Gly-Gly-Tyr	2.38 × 10^6^			
	Gly_2_-Trp-Gly_2_	2.20 × 10^4^	Sodium Phosphate 10 mM pH 7, co-binding with peptide-MV conjugates, 27 °C		[181]
	(Gly_2_-Trp-Gly_2_)_2_	5.00 × 10^5^			
	Asp_2_-(Gly_2_-Trp-Gly_2_)_3_-Asp_2_	4.70 × 10^6^			
MPnT	Lys-Ala-Ala	1.20 × 10^3^	Deuterium oxide, dihydrogenophosphate 25 mM pH 4.4	^1^H-NMR	[24]
		1.10 × 10^3^	Deuterium oxide, Phosphate buffer 200 mM pH 7.6		
	Lys-Lys-Leu-Val-Phe-Phe	1.41 × 10^4^			
		3.80 × 10^4^	Deuterium oxide, Sodium dihydrogenophosphate 25 mM pH 4.4		
	Lys-Thr-Thr-Lys	5.50 × 10^3^			
	Lys-Thr-Thr-Lys-Ser	4.20 × 10^3^			
	Gly-Arg-Gly-Gly	9.00 × 10^2^			
	Arg-Gly-Asp	1.20 × 10^3^			
		1.00 × 10^3^	Deuterium oxide unbuffered		
MPT	Lys-Ala-Ala	3.33 × 10^4^	Phosphate buffer 200 mM pH 7.6	Fluorescence Spectroscopy	[25]
	Lys-Leu-Val-Phe-Phe	5.00 × 10^4^			
	Lys-Lys-Leu-Val-Phe-Phe	2.50 × 10^5^			
	Lys-Lys-Leu-Val-Phe-Phe-Ala-Lys	1.43 × 10^5^			
	Lys-Lys-Lys-Lys	1 × 10^5^			
	Arg-Gly-Asp	1.16 × 10^4^			
MST	Lys-Ala-Ala	3.30 × 10^3^	Phosphate buffer 10 mM pH 7.6		
	Lys-Leu-Val-Phe-Phe	2.63 × 10^4^			
MCT	Lys-Ala-Ala	30	Phosphate buffer 10 mM pH 7.2		
PBI	Ala-Trp ^a^	3.72 × 10^9^	Acetonitrile:Methanol (9:1) 2:1 host guest complexes, 23 °C		[153]
CENMe_3_	Gly-Gly-Gly	2.10 × 10^2^	Water, 25 °C		[154]
	Gly-Trp-Gly	2.15 × 10^3^			
	Gly.Phe-Gly	1.7 × 10^3^			
	Gly-Gly-Phe	2.15 × 10^2^			
α-CDx	Gly-Leu	2.63 × 10^2^	Formic Acid 50%(*v*/*v*), 25 °C	ITC	[35]
	Gly-Val	2.82 × 10^2^			
β-CDx	Tyr-Ile-Gly-Ser-Arg	2.24 × 10^2^	Phosphate Buffer 0.01 M pH 7.0, 20 °C	Fluorescence Spectroscopy	[119]
	Tyr-Gly-Gly-Phe-Leu	1.23 × 10^2^			
	cyclic peptide -Asp-Phe-d-Pro-Asp-Phe-d-Pro-	2.20 × 10^2^	Bicarbonate buffer 0.2 M pH 9, 25 °C	ITC	[151]
DCv1	H3 (1–21) ^b^	2.11 × 10^5^	Tris Buffer 20 mM, pH 7.4, 25 °C		[159]
	PKP ^c^	2.68 × 10^5^			
	MBP ^d^	3.00 × 10^4^			
VD1	*N*-Ac_2_-l-Lys-d-Ala-d-Ala	1.02 × 10^5^	Water		[161]
	*N*-Ac_2_-l-Lys-d-Ala-d-Lac	3.19 × 10^4^			
GP3KKF	(d-Glu)_4_	2.37 × 10^4^	Bis Tris Buffer 1.5 mM pH 6.10	UV-Visible Spectroscopy	[164]
Pd6L4	Ac-Trp-Trp-Ala-NH_2_	>1.00 × 10^6^	Water, 20 °C		[179]
	Ac-Trp-Ala-Trp-NH_2_	2.50 × 10^5^			
	Ac-Ala-Trp-Trp-NH_2_	2.10 × 10^4^			
Fe^II^_8_L_6_ cage	Clavanin analogue ^e^	1.11 × 10^5^	Acetonitrile:water (1:1)		[180]
	Clavanin A ^a,f^	1.25 × 10^7^			

^a^ 1:2 host-guest stoichiometry. Overall binding constant in M^−2^. The overall binding constant corresponds to the product of the stepwise binding constants for the 1:1 and 1:2 complexes, i.e., *K*_11_.*K*_12_. ^b^ Ala-Arg-Thr-Lys-Gln-Thr-Ala-Arg-Lys-Ser-Thr-Gly-Gly-Lys-Ala-Pro-Arg-Lys-Gln-Leu-Ala. ^c^ Gly-Arg-Thr-Gly-Arg-Arg-Asn-Ser-Ile. ^d^ Ala-Pro-Arg-Thr-Pro-Gly-Gly-Arg-Arg. ^e^ Sequence of the analogue: Ser-Ser-Trp-Gly-His-Val-Gly-Lys-Tyr-Val-His-Gly-Trp-Ser-His-Val-Ser. ^f^ Val-Phe-Gln-Phe-Leu-Gly-Lys-Ile-Ile-His-His-Val-Gly-Asn-Phe-Val-His-Gly-Phe-Ser-His-Val-Phe.

## 5. Conclusions and Outlook

Despite the clear difficulty in the binding and recognition of the biological targets here presented, supramolecular systems begin to approach affinities and selectivities that are observable in nature, as is the case of protein–protein interactions, lock-and-key complementarity and the highly specific antigen–antibody recognition. Furthermore, many of the receptors explored here already possess the ability to directly modify or detect their target analytes, despite the majority of them presenting simple, modular structures. This is the case of *p*-sulfonatocalix[4]arene and cucurbit[n]urils, which can modulate the properties of proteins, such as cytochrome c and Casp-8, respectively. Additionally, some of these receptors present either an intrinsic colorimetric or fluorometric response in the presence of the respective ligands, e.g., the tweezers MPT and MCT, making them especially promising in sensing applications. Moreover, these characteristics are not the only ones that can be explored, seeing as the receptors can also be integrated in more complex structures, as synthetic binding pockets, with the addition of other groups for the creation of new functionalities.

The logical steps going forward are the continuous creation of new receptors, the eventual conjugation of several of these molecules for the multivalent binding of one complex analyte with several binding sights and smaller modifications and optimizations of the preexisting receptors, to enhance their selectivity and affinity towards one particular target ligand.

## Figures and Tables

**Figure 10:**
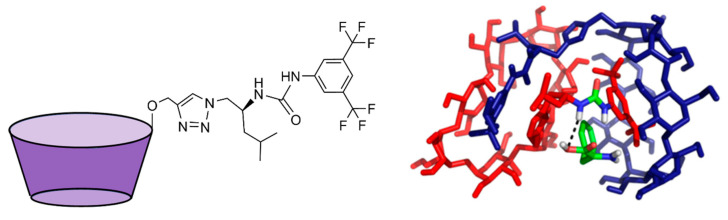
Structure of the receptor β-CDxU (**left**) and structure of the dimer that is formed upon complexation of l-Phe (**right**), reprinted with permission from [41]. Copyright (2017) Elsevier.

**Table 1:** Binding constants (*K*_a_) for the formation of host-guest complexes between amino acids and synthetic supramolecular receptors.

Host	Guest	*K*_a_ (M^−1^)	Conditions	Method	Ref.
SC4	l-Arginine	1.52 × 10^3^	Phosphate Buffer 10 mM pH 8, 20 °C	ITC	[21]
		1.7 × 10^3^	95% water/5% deuterium oxide pH 5	^1^H-NMR	[75]
		2.0 × 10^2^	95% water/5% deuterium oxide pH 1		
		1.55 × 10^3^	95% water/5% deuterium oxide pH 8, 20 °C		[22]
		6.40 × 10^3^	Deuterium oxide unbuffered, 25 °C		[88]
		1.30 × 10^4^	Deuterium oxide unbuffered, 25 °C		[86]
		2.8 × 10^4^	Acetate buffer 5 mM pD 4.5		
		3.30 × 10^2^	Deuterium oxide 40 mM phosphate buffer pD 7, 25 °C		[89]
	l-Lysine	7.4 × 10^2^	Phosphate Buffer 10 mM pH 8, 20 °C	ITC	[21]
		6.0 × 10^2^	95% water/5% Deuterium oxide pH 5	^1^H-NMR	[75]
		1.0 × 10^2^	95% water/5% Deuterium oxide pH 1		
		1.36 × 10^3^	95% water/5% Deuterium oxide pH 8, 20 °C		[22]
		<1 × 10^3^	Ammonium acetate buffer 10 mM, pH 6, 25 °C	Fluorescence Spectroscopy	[87]
		4.60 × 10^3^	Deuterium oxide unbuffered	^1^H-NMR	[86]
		3.90 × 10^3^	Acetate buffer 5 mM pD 4.5		
		5.20 × 10^2^	Phosphate buffer 40 mM, pH 7.4, 30 °C	ITC	[89]
	l-LysMe	4.00 × 10^3^			
	l-LysMe_2_	1.60 × 10^4^			
	l-LysMe_3_	3.70 × 10^4^			
	l-Histidine	5.00 × 10^2^	95% water/5% Deuterium oxide pH 8, 20 °C	^1^H-NMR	[22]
	l-Arginine	1.90 × 10^2^	Phosphate buffer pH 8, 20 °C		[21]
	l-Lysine	90			
	l-Arginine	3.50 × 10^2^			
SC4-Ar	l-Lysine	4.2 × 10^2^	Phosphate buffer 40 mM pD 7.0, 25 °C		[90]
	l-LysMe_3_	6.4 × 10^4^			
C4Pyr	l-Lysine	1.40 × 10^2^	Phosphate Buffer pH 8, 20 °C		
	l-Glutamate	1.76 × 10^3^	Water pH 6	Fluorescence Spectroscopy	[52]
	l-Aspartate	1.16 × 10^3^			
P5 Bodipy	l-Asparagine	2.5 × 10^5^	Dimethyl formamide/water 1:1, 25 °C		[95]
CP5	l-Arginine	5.24 × 10^3^	HEPES buffer pH 7.4, 25 °C		[94]
		5.90 × 10^3^	Deuterium oxide pD 7.2, 25 °C	^1^H-NMR	[26]
	l-Lysine	1.12 × 10^3^	HEPES buffer pH 7.4, 25 °C	Fluorescence Spectroscopy	[94]
		1.80 × 10^3^	Deuterium oxide pD 7.2, 25 °C	^1^H-NMR	[26]
	l-Histidine	1.50 × 10^3^			
	TriMe-Lys	1.30 × 10^3^			
	Ac-Lys	1.90 × 10^2^			
PDA6	l-Glutamate	1.00 × 10^6^	Water pH 6, 25 °C	Fluorescence Spectroscopy	[51]
	l-Aspartate	9.80 × 10^5^			
MPnT	l-Lysine	1.40 × 10^3^	Deuterium oxide, Sodium Dihydrogenophosphate 25 mM pH 4.4	^1^H-NMR	[24]
	Ac-His-OMe	7.00 × 10^2^			
	Ac-Lys-OMe	4.40 × 10^3^	Deuterium oxide unbuffered		
		2.30 × 10^4^			
	l-Lysine	1.14 × 10^3^	Phosphate buffer 200 mM pH 7.6	Fluorescence Spectroscopy	[25]
MPT	l-Lysine	4.76 × 10^4^			
	Ac-Arg-OMe	1.67 × 10^4^			
	Ac-Lys-OMe	5.88 × 10^4^			
	Ac-Arg-OMe	5.00 × 10^4^	Phosphate buffer 10 mM pH 7.6		
	Ac-Lys-OMe	1.11 × 10^5^			
MST	l-Lysine	4.41 × 10^3^			
	l-Arginine	1.43 × 10^3^			
	Ac-Arg-OMe	1.30 × 10^4^	Phosphate buffer 10 mM pH 7.2		
	Ac-Lys-OMe	5.26 × 10^4^			
MCT	l-Lysine	8.60 × 10^2^	Phosphate buffer 10 mM pH 7.2		
	l-Arginine	1.64 × 10^3^			
	Ac-Arg-OMe	3.56 × 10^3^			
	Ac-Lys-OMe	1.56 × 10^3^			
	Ac-Lys-OMe	4.42 × 10^3^	Phosphate buffer 200 mM pH 7.6		
CEAT	Lys-OMe	3.16 × 10^4^	Methanol		[118]
		3.98 × 10^4^	Water with 1.6% Methanol		
		7.0 × 10^1^	HEPES buffer 50 mM pH 7.5 with 1.6% Methanol		
CB6	l-Lysine	1.10 × 10^4^	Deuterium oxide 0.1 M NaCl, 25 °C	^1^H-NMR	[101]
	Glycine	4.70 × 10^3^	Aqueous Formic Acid 50%(*v*/*v*), 25 °C	ITC	[35]
	l-Alanine	1.00 × 10^3^			
	l-Valine	1.40 × 10^3^			
	l-Phenylalanine	1.40 × 10^3^			
CB7	l-Phenylalanine	1.80 × 10^6^	Water, 25 °C		[36]
		1.80 × 10^5^	Phosphate Buffer pH 7, 25 °C		[102]
		1.20 × 10^6^	Phosphoric acid buffer pH 2, 25 °C		
		1.50 × 10^5^	Water, 25 °C	^1^H-NMR	[44]
		8.20 × 10^5^	nd	UV-Visible Spectroscopy	
	l-Tyrosine	2.30 × 10^5^			
		1.60 × 10^4^	Phosphate Buffer pH 7, 25 °C	ITC	[102]
		1.80 × 10^5^	Phosphoric acid buffer pH 2, 25 °C		
		2.40 × 10^4^	Ammonium Acetate Buffer pH 6, 30 °C	Fluorescence Spectroscopy	[45]
		2.20 × 10^4^		ITC	
	l-Tryptophan	3.70 × 10^5^	nd	UV-Visible Spectroscopy	[44]
		1.20 × 10^3^	Phosphate Buffer pH 7, 25 °C	ITC	[102]
		7.40 × 10^3^	Phosphoric acid buffer pH 2, 25 °C		
		1.60 × 10^3^	Ammonium Acetate Buffer 10 mM pH 6, 25 °C	Fluorescence Spectroscopy	[45]
		1.90 × 10^3^	Ammonium Acetate Buffer 10 mM pH 6, 30 °C	ITC	
	l-Lysine	8.70 × 10^2^	Ammonium Acetate Buffer 10 mM pH 6, 25 °C	Fluorescence Spectroscopy	
		8.00 × 10^2^	Ammonium Acetate Buffer 10 mM pH 6, 30 °C	ITC	
		2.10 × 10^2^	Phosphate Buffer pH 7, 25 °C		[102]
		3.10 × 10^5^	Phosphoric acid buffer pH 2, 25 °C		
		5.30 × 10^2^	Sodium Acetate Buffer 50 mM, deuterium oxide pD 4.7, 25 °C	^1^H-NMR	[101]
	Lys-NMe	1.80 × 10^3^			
	Lys-NMe_2_	6.00 × 10^4^			
	Lys-NMe_3_	1.90 × 10^6^			
	l-Arginine	3.10 × 10^2^	Ammonium Acetate Buffer 10 mM pH 6, 25 °C	Fluorescence Spectroscopy	[45]
		1.40 × 10^5^	Phosphoric acid buffer pH 2, 25 °C	ITC	[102]
		3.27 × 10^2^	Ammonium Acetate Buffer 10 mM pH 6, 30 °C		[45]
	l-Histidine	4.00 × 10^2^	Ammonium Acetate Buffer 10 mM pH 6, 25 °C	Fluorescence Spectroscopy	
		2.40 × 10^3^	Phosphoric acid buffer pH 2, 25 °C	ITC	[102]
CB8	l-Tyrosine ^a^	<1.00 × 10^3^	Sodium Phosphate 10 mM pH 7 CB8-AA_2_, 27 °C	ITC	[110]
	l-Phenylalanine ^a^	1.10 × 10^8^			
	l-Tryptophan ^a^	6.90 × 10^7^			
	l-Tyrosine	2.20 × 10^3^	Sodium Phosphate 10 mM pH 7, co-binding with MV, 27 °C		[37]
	l-Phenylalanine	5.30 × 10^3^			
	l-Tryptophan	4.30 × 10^4^			
	l-Trp-OMe	6.30 × 10^4^			
	l-Tryptophan	4.20 × 10^5^	Co-binding with DPT	UV-Visible Spectroscopy	[38]
		3.40 × 10^4^	Sodium Phosphate 10 mM pH 7, co-binding with MBBI, 27 °C	ITC	[39]
CB6Ar	l-Phenylalanine	4.20 × 10^4^	Sodium Acetate 50 mM pH 4.74, 22 °C	Fluorescence Spectroscopy	[40]
	l-Tyrosine	5.70 × 10^4^			
	l-Tryptophan	3.20 × 10^6^			
α-CDx	Glycine	5.60 × 10^2^	Aqueous Formic Acid 50%(*v*/*v*), 25 °C	ITC	[35]
	l-Alanine	1.12 × 10^3^			
	l-Valine	1.62 × 10^3^			
	l-Phenylalanine	2.6 × 10^2^			
β-CDx	l-Tyrosine	5.0 × 10^1^	Phosphate Buffer 0.01 M pH 7.0, 20 °C	Fluorescence Spectroscopy	[119]
β-CDU	l-Tryptophan	5.24 × 10^4^	Phosphate buffer 50 mM, 25 °C	ITC	[120]
	l-Phenylalanine	2.24 × 10^4^			
	l-Tyrosine	1.33 × 10^4^			
	l-Alanine	5.00 × 10^3^			
	l-Serine	1.97 × 10^4^			
CPQ	l-Tryptophan	1.00 × 10^3^	Phosphate buffer 50 mM pH 7, 25 °C	^1^H-NMR	[116]
	l-Tyrosine	4.54 × 10^2^			
	l-Phenylalanine	1.06 × 10^2^			
Cav[(CH_2_)_2_ CH_3_, CH_2_CH_3_]	*N*-Me-Leucine·HCl	1.70 × 10^5^	Methanol, 20 °C	ITC	[115]
	*N*-Me-Alanine·HCl	1.40 × 10^5^			
	Proline·HCl	1.20 × 10^4^			
	Threonine·HCl	2.00 × 10^4^			
	Alanine·HCl	1.10 × 10^4^			
	Tyrosine·HCl	1.10 × 10^4^			
	Cysteine·HCl	7.30 × 10^3^			
	*N*-Me-Lysine·HCl 1:1	1.07 × 10^6^	Methanol, 20 °C (amino acid:cavitand)		
	*N*-Me-Lysine·HCl 1:2	1.45 × 10^3^			
Cav[(CH_2_)_2_ CH_3_Py^+^Cl^−^, CH_2_CH_3_]	*N*-Me-Glycine-methyl ester·HCl	6.80 × 10^4^	Methanol, 20 °C		
		3.43 × 10^3^	Water, 20 °C		
		1.89 × 10^3^	Phosphate Buffer Saline 0.1 M pH 7, 20 °C		
	Sarcosine	1.02 × 10^3^	Water, 20 °C		
	*N*-Me-Lysine·HCl	1.49 × 10^3^			
		1.13 × 10^3^	Phosphate Buffer Saline 0.1 M pH 7, 20 °C		
TeCPh	l-Tryptophan	1.21 × 10^3^	Phosphate Buffer 10 mM pH 7.4, 25 °C		[42]
OA (R,R)-1	*N*-Ac-d-Aspartate	1.62 × 10^4^	Tetramethylammonium chloride 0.1 M, 20 °C	pH-metric titrations	[53]
	*N*-Ac-d-Glutamate	3.31 × 10^3^			
OA (S,S,S,S)-2	*N*-Ac-l-Aspartate	3.40 × 10^2^			
	*N*-Ac-l-Glutamate	6.30 × 10^2^			
GP3cat	*N*-Ac-l-Alanine	2.10 × 10^3^	1:1 90% water/10% Dimethyl sulfoxide	ITC	[59]
	*N*-Ac-l-Glutamate	1.52 × 10^6^	2:1 90% water/10% Dimethyl sulfoxide		

^a^ 1:2 host-guest stoichiometry. Overall binding constant in M^−2^. The overall binding constant corresponds to the product of the stepwise binding constants for the 1:1 and 1:2 complexes, i.e., *K*_11_.*K*_12_.

## Data Availability

No new data were created or analyzed in this study. Data sharing is not applicable to this article.
